# Spatially refined satellite gravimetry captures human signatures in global terrestrial water storage trends

**DOI:** 10.1073/pnas.2600775123

**Published:** 2026-09-14

**Authors:** Mary Michael O’Neill, Matthew Rodell, Bryant D. Loomis

**Affiliations:** ^a^ Hydrological Sciences Laboratory, Earth Sciences Division, National Aeronautics and Space Administration, Goddard Space Flight Center, Greenbelt, MD 20771; ^b^ Earth System Science Interdisciplinary Center, University of Maryland, College Park, MD 20742; ^c^ Earth Sciences Division, National Aeronautics and Space Administration, Goddard Space Flight Center, Greenbelt, MD 20771; ^d^ Geodesy and Geophysics Laboratory, Earth Sciences Division, National Aeronautics and Space Administration, Goddard Space Flight Center, Greenbelt, MD 20771

**Keywords:** gravity, terrestrial water storage, groundwater

## Abstract

Human activities have directly altered the water cycle through water management, aquifer pumping, agricultural irrigation, and land use change. Although satellite gravimetry has transformed global hydrological research, its coarse resolution limits attribution of freshwater change to human activities at many management-relevant scales. Here we assessed global terrestrial water storage (TWS) trends from April 2002 to November 2025 using “stacked” regression of Level-1B intersatellite ranging data, which leverages temporal information and variability to dramatically improve effective spatial resolution relative to standard approaches. We combined this refined product with rigorous uncertainty analysis, autocorrelation-robust geostatistical methods, and literature assessment to evaluate TWS trend associations with land and water use, climate variability, and glacial mass loss. We identified 40 TWS trend hotspots exhibiting significant spatial associations with anthropogenic drivers, including groundwater and surface-water irrigation, rainfed agriculture, deforestation, and reservoir impoundment. Across these regions, cumulative TWS losses (3,122 Gt) substantially exceeded gains (2,432 Gt). Compared with traditional regression of monthly mascons, our approach yielded regional trend magnitudes that are on average 33% larger, revealing that global freshwater depletion, particularly from groundwater pumping, is considerably more acute than previously estimated. Multivariate regression models show that humans account for a significant share of the spatial variability in TWS trends on every nonpolar continent except Australia. We detected localized TWS gains linked to rainfed agriculture, surface water irrigation, and reservoir filling that were unresolved in earlier gravimetric studies. The methodology provides a foundation for future gravity missions to independently track decadal freshwater change with unprecedented spatial fidelity.

## Significance

It is well known that human activities directly impact the water cycle and the distribution of water resources, but quantifying such effects from space is challenging. By directly estimating mass change rates from raw satellite range observations, we reduced signal loss and mapped substantially finer-resolution global terrestrial water storage trends. Herein, we have identified trends that we attribute to human activities in 40 regions around the world. Anthropogenic land and water use was significantly associated with strong TWS trends in every nonpolar continent except Australia, and groundwater depletion linked to agriculture was particularly severe.

Earth’s freshwater stores naturally fluctuate, but, in many regions, changes caused by human activities have surpassed natural variability (1). Appropriation of water for crops, pasture, industry, and municipalities is now a fundamental component of the water cycle (2, 3) which is exacerbated by climate change (4). Estimates of global total freshwater withdrawals range from 3,800 to 5,000 Gt y^−1^ (2), and unsustainable practices have resulted in pervasive water scarcity (5–7). Identifying and accurately quantifying anthropogenic, long-term changes in freshwater resources is critical for implementing strategies to ensure water and food security.

Launched in 2002 and 2018, NASA’s Gravity Recovery and Climate Experiment (GRACE) and its Follow-On successor (GRACE-FO) have used precise satellite-to-satellite range measurements to quantify tiny shifts in Earth’s gravity field, from which are inferred redistributions of mass in the atmosphere, oceans, and continents (8, 9). The aggregate mass of all water stored in the ground and land surface, terrestrial water storage (TWS) is crucial as a holistic indicator of freshwater availability and an Essential Climate Variable (10, 11). GRACE/FO observations of TWS anomalies have underpinned hundreds of hydrological studies, resolving both natural climate variability and redistribution of water by agriculture, industry, and municipalities (10, 12). Multidecadal TWS trends that implicate water and food security have received particular scrutiny (1, 13).

However, state-of-the-art mass concentration (“mascon”) retrievals of monthly GRACE/FO TWS anomalies (14–16) cannot effectively resolve spatial scales smaller than ~150,000 km^2^ without data assimilation (17), scale factors (18, 19), or a high tolerance for uncertainty (20). Traditional regression of monthly mascons smooth out local mass trends (21), limiting the value of GRACE/FO for watershed-scale applications, such as pinpointing exact locations of highest freshwater consumption.

In this study, we used an innovative GRACE/FO processing method to spatially refine global TWS trends. Recently, a processing technique was introduced that improves by up to an order of magnitude the spatial resolution of multidecadal TWS trends inferred from GRACE/FO data (22–25). By regressing many years of “stacked” Level-1B intersatellite ranging data, substantially more fine-scale information is retained (*Materials and Methods* and *SI Appendix*, Fig. S1). This method has been shown to improve GRACE/FO-derived mass change estimates of Antarctic and Greenland ice sheets, relative to altimetry measurements (22).

**Fig. 1.**
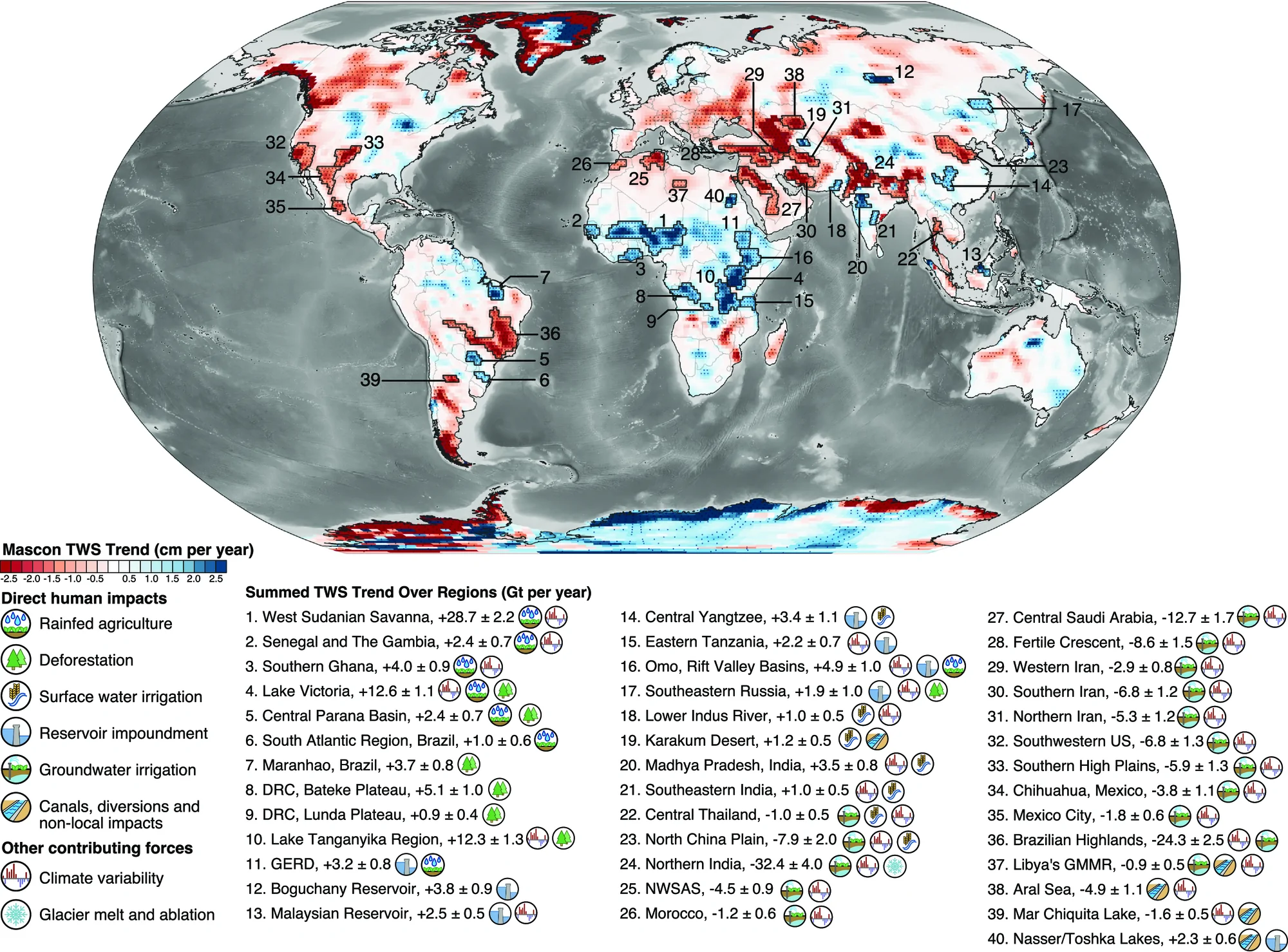
Human activities impact TWS trends across the globe. Global trends in TWS from GRACE (April 2002 to August 2016) and GRACE-FO (June 2018 to November 2025) across 1° equal area mascons (Dataset S1 and *SI Appendix*, Table S2). Regions 1 to 38 are areas where trend magnitude exceeds uncertainty and where anthropogenic impacts to the water cycle are spatially associated with TWS trend. Icons refer to local human activities and other possible contributing drivers (see *SI Appendix* for details). Uncertainty bounds incorporate error in glacial isostatic adjustment and gravimetry solution noise.

**Fig. 2.**
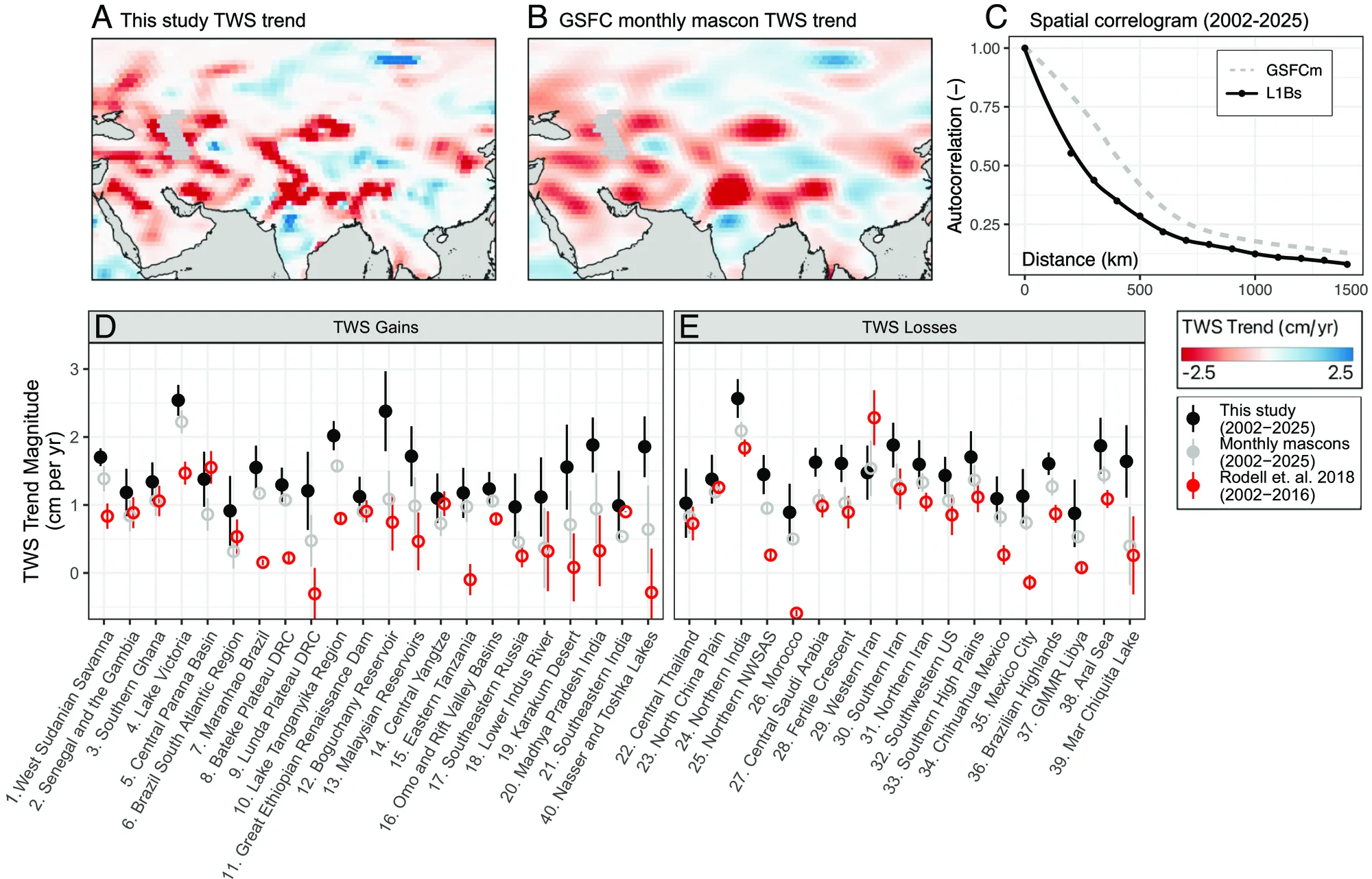
Previous studies and standard processing methods may underestimate TWS trends. Spatial patterns of GRACE-derived TWS trends are compared between this study, solutions from previous publications, and common monthly mascon regression techniques. TWS trends are shown across southern Asia for (*A*) this study, using high-resolution regression (April, 2002 to November, 2025), and (*B*) regression of GSFC monthly mascon (Level-3) regression (15) (April, 2002 to November, 2025). (*C*) Spatial autocorrelation (Moran’s I) at various spatial lags from TWS trends found using the Level-1B regression, along with standard regression of GSFC monthly mascons (15) linearly interpolated to our mascon locations. Mean TWS gains (*D*) and losses (*E*) over 40 study regions, calculated from this study (black), an average of three popular monthly mascon solutions (gray), and a highly cited study of 2002–2016 Level-3 TWS trends (1). Averages were found using area-weighted mean. Uncertainty bars on the mean Level-3 solutions represent the SD of three monthly mascon products (*SI Appendix*, Table S2).

We used the Level-1B regression approach to evaluate global, nonpolar TWS trends from April 2002 through November 2025 across 1-arc-degree (~111 km) equal-area mascons (Fig. 1), producing a global TWS trend map at 5 to 10 times the spatial resolution of traditional monthly mascons (Fig. 2 and *SI Appendix*, Fig. S1 and Table S1). We then performed a rigorous uncertainty analysis (*SI Appendix*, Fig. S2 and Table S2) to identify clusters of mascons where trends exceed expected solution noise and glacial isostatic adjustment uncertainty, revealing regional-scale TWS trend hotspots. To investigate the drivers of these hotspots, we applied spatial autoregressive models to evaluate the continental-scale relationships between TWS trends and a suite of climate (26, 27) and anthropogenic variables (28–34) (*SI Appendix*, Figs. S3 and S4 and Tables S3–S5), quantifying the relative influence of each variable across each nonpolar continent. Finally, we used statistical local indicators of spatial association, paired with literature review, to determine which TWS trend hotspots are consistent with these climate or anthropogenic relationships, identifying 40 regions where human activities are implicated as drivers of decadal TWS change. The Level-1B regression approach forgoes temporal variability in favor of static regression coefficients, but the resulting TWS trend map represents the upper bounds of secular signal detection achievable with GRACE and GRACE-FO.

## Spatially Refined Global TWS Trends

GRACE/FO TWS fields are strongly spatially autocorrelated not only because of genuine climate, hydrologic, and topographic controls, but also because the gravity inversion and filtering procedures introduce artificial smoothing and signal leakage, producing nonphysical spatial dependence that should be minimized (20). Continental-scale patterns of TWS change that we derived from Level-1B regression (Fig. 1) broadly agree with those previously observed and based on the standard monthly mascons, including TWS gains in Africa’s Rift Valley, the Western Sahel, and Southeast Asia, and widespread TWS losses in the Middle East and Northern Africa (MENA) regions, northern India, the western United States and southeastern Brazil (1, 35–38). However, the high-resolution approach sharpens the picture substantially, reducing smoothed or attenuated signal and allowing detection of more localized trends.

To illustrate, Fig. 2 *A* and *B* compare the new results with conventional TWS trends from monthly mascons over the Middle East and southern Asia. The latter map, which we derived via linear regression of monthly mass anomaly mascons (15), exhibits similar large scale patterns but lacks fine detail. For consistency with spatial resolution definitions in much of the GRACE literature (15), we define the spatial resolution as the Gaussian smoothing radius that best models the distance-decay of a synthetic impulse response (*SI Appendix*, Fig. S1). This analysis results in best-fit Gaussian smoothing radii of 100 to 110 km for the L1B stacked solutions and 235 to 350 km for the monthly mascon regression, which corresponds to 5 to 10 factor improvements for the stacked estimates in terms of spatial area. Visually, the improved spatial resolution of the new map is dramatic (~12,000 km^2^ vs. >120,000 km^2^ at mid-latitudes) (Fig. 2). For additional comparisons between contemporaneous maps, we computed 2002–2025 trends via linear regression of other popular GRACE/FO monthly mascon products (15, 16, 39), with similar results (*SI Appendix*, Figs. S5 and S6). Fig. 2*C* illustrates the statistical significance of this visual “smoothing” improvement using correlograms of global TWS trends derived from monthly mascon regression and our solution. The Level-1B regression exhibits both a markedly steeper initial decline in spatial autocorrelation and a shorter autocorrelation length (distance at which autocorrelation becomes insignificant) (Fig. 2*C*). Further, in regions experiencing significant TWS change, L1B regression shows much more intense trend magnitudes (Fig. 2 *D* and *E* discussed below).

L1B regression mascons exceeding expected product noise uncertainty accounted for roughly 22% of global land area and were clustered into 94 distinct regions (*SI Appendix*, Fig. S2). TWS losses from mascons with significant negative trends totaled 404 Gt y^−1^ (13% of land area), while significant gains summed to 176 Gt y^−1^ (9% of land area). The net flux of terrestrial water across all land mascons (−270 Gt y^−1^) corresponds to a barystatic sea level change of −1.9 mm y^−1^, which is congruent with recent global mean sea level budget closure (40). Attribution of the L1B-stacked TWS trend hotspots to climatic and anthropogenic agents is discussed below.

## Continental-Scale Associations Between TWS Trends and Human Activity

The refinement shown in Fig. 2 is important when evaluating spatial relationships, because autocorrelation reduces the effective degrees of freedom in gridded fields, artificially inflating test statistics (41). To posit climate or human drivers of TWS trends, we first constructed multivariate regression models to identify significant continental-scale spatial relationships between TWS trends (the dependent variable) and gridded predictors. Independent human-influenced predictors included percentage of deforested land area (28), percentage of groundwater-irrigated or surface-water irrigated land area (31), percentage of rainfed cropland area (29), and summed 21st century reservoir storage impoundment (34). Predictors related to climate variability (we did not separate anthropogenic and natural climate variability), included anomalies (relative to 40-y mean), and trends in precipitation and vapor pressure deficit (27), as well as 21st century glacial mass change (26). All were aggregated to the mascon scale (*SI Appendix*, Figs. S3 and S4) and standardized prior to regression, enabling direct comparisons of influence. Because GRACE-derived TWS trends exhibit strong spatial dependence that violates the independence assumption of standard ordinary least squares, we employed spatial autoregressive models. These models yield unbiased coefficient estimates and more reliable inference about the relative influence of climatic and anthropogenic predictors, and they have been widely applied across geophysical and econometric disciplines (42–45).

**Fig. 3.**
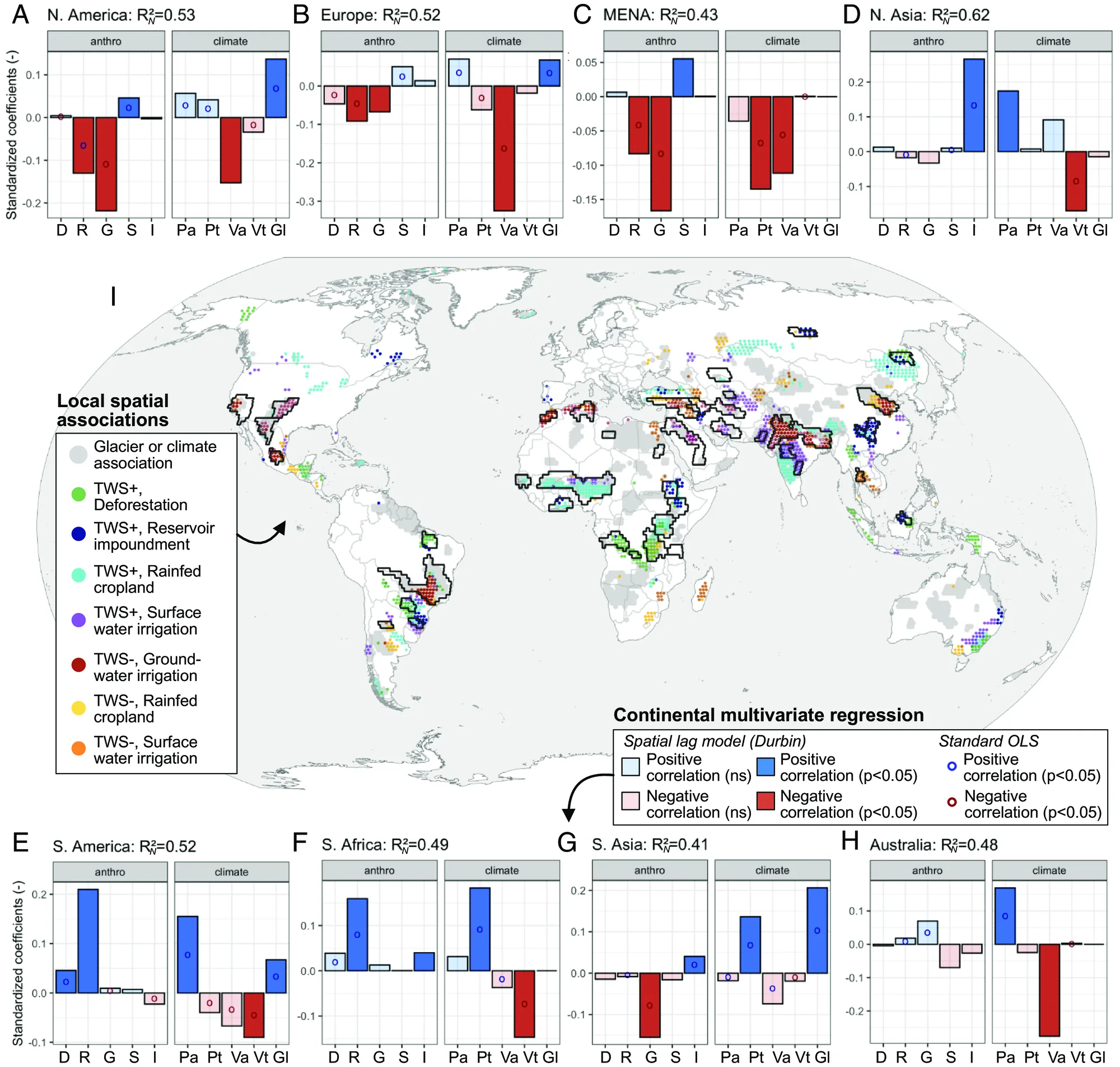
Results from continental-scale multivariate regression and local bivariate spatial association, used for TWS trend attribution. (*A*–*H*) Standardized coefficients from spatial Durbin model for unlagged independent variables, deforested land area (*D*), rainfed cropland area (R), groundwater-irrigated cropland area (G), surface water-irrigated cropland area (S), reservoir impoundment (I), precipitation anomaly (Pa) and trend (Pt), vapor pressure deficit anomaly (Va) and trend (Vt), and glacial mass loss (Gl). Blue (red) bars indicate positive (negative) associations, with opacity indicating significance (darker colors mean *P* < 0.05). Circle annotations indicate ordinary least squares significance. Results are shown for models run over subpolar North America (*A*), Europe (*B*), Northern Africa and the Middle East (*C*), subpolar northern Asia (*D*), South America (*E*), sub-Saharan Africa (*F*), southern and maritime Asia (*G*), and Australasia (*H*). (*I*) Significant local Lee’s *L* for bivariate TWS trend and independent predictor pairs observed from continental spatial lag models. *R_N_^2^* represents Nagelkerke’s pseudo-R^2^ (46), which signifies relative model performance (1 being perfect). *R_N_^2^* ranges are typically lower than coefficient of determination and should not be interpreted as explained variance.

Spatial models evaluated at the continental scale indicate that human land and water use significantly influence global TWS trend spatial structure (Fig. 3). Fig. 3 *A*–*H* show standardized coefficients for each variable, which express the change in the response variable, in units of its own SD, associated with a one SD increase in a given predictor. In every nonpolar continental region except Australia, a human activity held significant influence on spatially variable TWS trends. An anthropogenic variable had the strongest relative influence on TWS trends (highest magnitude coefficient) in North America, the MENA region, Northern Asia, and South America. Primary controls on TWS trends include negative associations with groundwater-irrigated land area and vapor pressure deficit, and positive associations with precipitation, surface water irrigated land area and glacial mass loss. Relationships between TWS trends and rainfed cropland area varied by continent, and deforestation was only a significant predictor (positively correlated) in South America. Fig. 3 *A*–*H* also indicates which variables were significant using standard ordinary least squares regression; spatial autoregressive modeling typically yields more conservative results.

We applied local indicators of spatial association (LISA) (43, 47) to identify hotspots where TWS trends and individual predictor variables exhibited statistically significant spatial co-occurrence consistent with the continental-scale relationships (Fig. 3*I*). Significant TWS trends were attributed to human activities where LISA covariance, continental-scale multivariate results, and existing literature collectively supported the relationship. We added a handful of regions that statistical methods missed due to nonlocal human influences such as upstream bifurcations, diversions, and dams. Supporting regional statistics are shown in *SI Appendix*, Tables S2–S5, while Fig. 1 shows attribution categories.

Of the original 94 significant TWS trend hotspots (*SI Appendix*, Fig. S2, excluding polar regions and ice sheets), we identified 40 study regions where strong TWS trends were demonstrably associated with human land or water usage (Fig. 1). The 40 regions shown in Fig. 1 range in size from ~74,000 km^2^ to over 1,500,000 km^2^. During the study period, TWS losses and gains in these regions totaled −3,122 Gt and 2,432 Gt, the former exceeding the volume of Lake Victoria. Traditional regression of monthly mascon products over our study regions underestimated TWS trend magnitudes by an average of 33%, and by 52% for the seven regions smaller than 100,000 km^2^, demonstrating that the difference in resolution becomes more important at smaller scales (*SI Appendix*, Fig. S7 and Table S6). Comparisons with a highly cited global TWS trend map from Rodell et al. (1) (2002–2016) were even more stark (Fig. 2*D*), suggesting that many of these trends persist or could have intensified in the last decade. In short, global TWS trends are more acute than previously known.

Below, we discuss the 40 regions identified from the new high-resolution TWS trend map. We compare our findings with previous literature and note where the observed relationships between storage trends and important anthropogenic activities were unknown, poorly constrained, underestimated, or undetectable by gravimetry.

## Rainfed Agriculture Enhances Groundwater Recharge

Spatial autoregressive models and local spatial indicators suggest that rainfed cropland is typically only associated with negative TWS trends where it coexists with groundwater-irrigated cropland (Fig. 3*I*); elsewhere, correlations with TWS trends are largely positive. In humid and semiarid grasslands of equatorial Africa, swaths of native savanna have been converted to millet (48). Here, spatial autoregressive models indicate that rainfed cropland area is a significant driver of TWS trend spatial structure (positively correlated), nearly as influential a predictor as precipitation trends (Fig. 3). Across sub-Saharan Africa, spatial patterns of rainfed cropland (29) resemble positive TWS trends calculated using the Level-1B regression approach (*SI Appendix*, Fig. S8).

Within Regions 1 through 3, TWS trends totaled 35.0 ± 3.7 Gt y^−1^ despite lower-than-average precipitation during the GRACE/FO period. The largest of these, the West Sudanian Savanna (Region 1, 28.7 ± 2.2 Gt y^−1^), is 17% rainfed cropland and has experienced a 14% reduction in native grassland or shrubland area since 1960 (*SI Appendix*, Table S3) (28). Positive TWS trends here and in Senegal and the Gambia (Region 2, 2.4 ± 0.7 Gt y^−1^) and southern Ghana (Region 3, 4.0 ± 0.9 Gt y^−1^), may be impacted by precipitation trends (roughly 5 to 10 mm yr^-1^) (*SI Appendix*, Table S4). Positive TWS trends around Lake Victoria (Region 4, 12.6 ± 1.1 Gt y^−1^) can partially be attributed to rising lake water levels and record-breaking El Niño rains in 2019 and 2020 (49), as well as regulation of the Nalubaale Dam (50). However, rainfed cropland area is significantly colocated with TWS increase (Fig. 3), and local land use practices have notably impacted influx streamflow (51). Rainfed agriculture comprises between 15% and 18% of these regions (*SI Appendix*, Table S3).

Similarly, in South America, there appears to be a relationship between loss of native vegetation and positive TWS trends in forests and subtropical shrublands, particularly along the arc of deforestation and soybean expansion (52). The presence of rainfed agriculture is the most significant predictor of TWS trend in the multivariate analysis in South America (Fig. 3*E*). Local spatial associations (Fig. 3*I*) occur in the central Parana Basin (Region 5, 2.4 ± 0.7 Gt y^−1^, 25% rainfed cropland area) and the Brazil South Atlantic Region (Region 6, 1.0 ± 0.6 Gt y^−1^, 32%), despite historically negative precipitation trends and high vapor pressure deficit (*SI Appendix*, Tables S4 and S5). Deforested land area since 1960 is significantly positively related to TWS trend at the continental scale (Fig. 3*E*), with Maranhao, Brazil (Region 7) experiencing a water storage increase of 3.7 ± 0.8 Gt y^−1^ and reduction in forested area of 15%. Although the continental-scale relationship between these two variables is insignificant (albeit positive) in Africa, deforestation is the only variable that is colocated with TWS trends in the southern plateau regions of the Democratic Republic of the Congo (Fig. 3*I*; Region 8, 5.1 ± 1.0 Gt y^−1^, 24% deforested; Region 9, 0.9 ± 0.4 Gt y^−1^, 54% deforested). The Lake Tanganyika Region (Region 10) also experienced significant deforestation (14%) since 1960 and a GRACE/FO TWS trend of 12.3 ± 1.3 Gt y^−1^, compounded by a strong increasing trend in precipitation (11 mm yr^-1^).

A physical explanation is that rainfed legumes and cereals transpire less and access shallower groundwater stores than native vegetation, owing to lower root/shoot ratios (53) and inherent water use efficiency (54, 55). Indigenous soil and water conservation practices (zaï pits, half-moons, and stone bunds across Burkina Faso and Niger) further enhance infiltration and reduce runoff (56), directing rainfall into shallow aquifers. Comparable dynamics are observed in South America, where cropland expansion is associated with raised water tables in the Chaco Plains (57), increased water yield in the Amazon (58–60), and an accelerated Brazilian water cycle (61). Previous large-scale studies have suggested, but never explicitly shown, that 20th-century land clearing for rainfed Sahelian millet raised the water table through this mechanism (48, 62), and that land use change has contributed to large-scale TWS trends in the equatorial and semiarid tropics (63).

However, both precipitation and vapor pressure deficit exert strong influence on TWS trend spatial patterns (Fig. 3), with robust correlations globally and particularly in sub-Saharan Africa (Pearson’s r = 0.63 to 0.74, *SI Appendix*, Fig. S9). Regional-scale climate trends are statistically significant across Regions 1 to 3 (*SI Appendix*, Tables S4 and S5), and their spatial patterns largely align with TWS. For example, many mascons within Region 1 experience both increasing precipitation and decreasing vapor pressure deficit despite no significant surrounding trend (*SI Appendix*, Fig. S4), suggesting coupled land–atmosphere feedbacks. Observational evidence from the Sahel demonstrates that vegetation greenness anomalies are associated with enhanced evapotranspiration, moisture recycling, and convective rainfall (64), while Amazonian deforestation has been shown to reduce local dry-season precipitation and increase nonlocal wet-season precipitation through mesoscale circulation (65). Given the strong correlation between precipitation and vapor pressure deficit trends (*SI Appendix*, Fig. S9), our spatial autoregressive model likely cannot fully disentangle their independent contributions to TWS. Variance inflation factor (VIF) calculations suggest multicollinearity is not a formal statistical concern (*SI Appendix*, Table S7), but isolating the relative influence of precipitation vs. vapor pressure deficit exceeds what this technique alone can resolve.

## Reservoir Impoundments Locally Increase Freshwater Storage

Gravimetry observations also captured surface water withdrawals and displacements for agricultural, domestic, and industrial uses, which total 2,800 Gt y^−1^ globally (33). Dam construction has surged during the GRACE/FO period, with 450 large reservoirs being filled and global storage increasing by 746 Gt to over 6,800 Gt (33). Earlier attempts to monitor reservoir impoundment using satellite gravimetry (66) were hindered by the scale mismatch and prohibitively high uncertainty (67). Level-1B regression overcomes these challenges. At the continental scale, we found that relationships between TWS trends and reservoirs were significantly positive in Northern Asia, sub-Saharan Africa, and Southeastern Asia (Fig. 3). Several local spatial associations are notable (Fig. 3*I*).

Inaugurated in 2025 on the Blue Nile, the Grand Ethiopian Renaissance Dam (GERD) is Africa’s largest hydroelectric facility, built to expand Ethiopian power generation but contentious among downstream Egypt and Sudan over its effects on Nile water availability (68). The reservoir at 74 Gt capacity, filled between 2020 and 2025, entirely explains the TWS trend in Region 11 (3.2 ± 0.8 Gt y^−1^), which sum to 75 ± 19.4 Gt gained during the observation period.

**Fig. 4.**
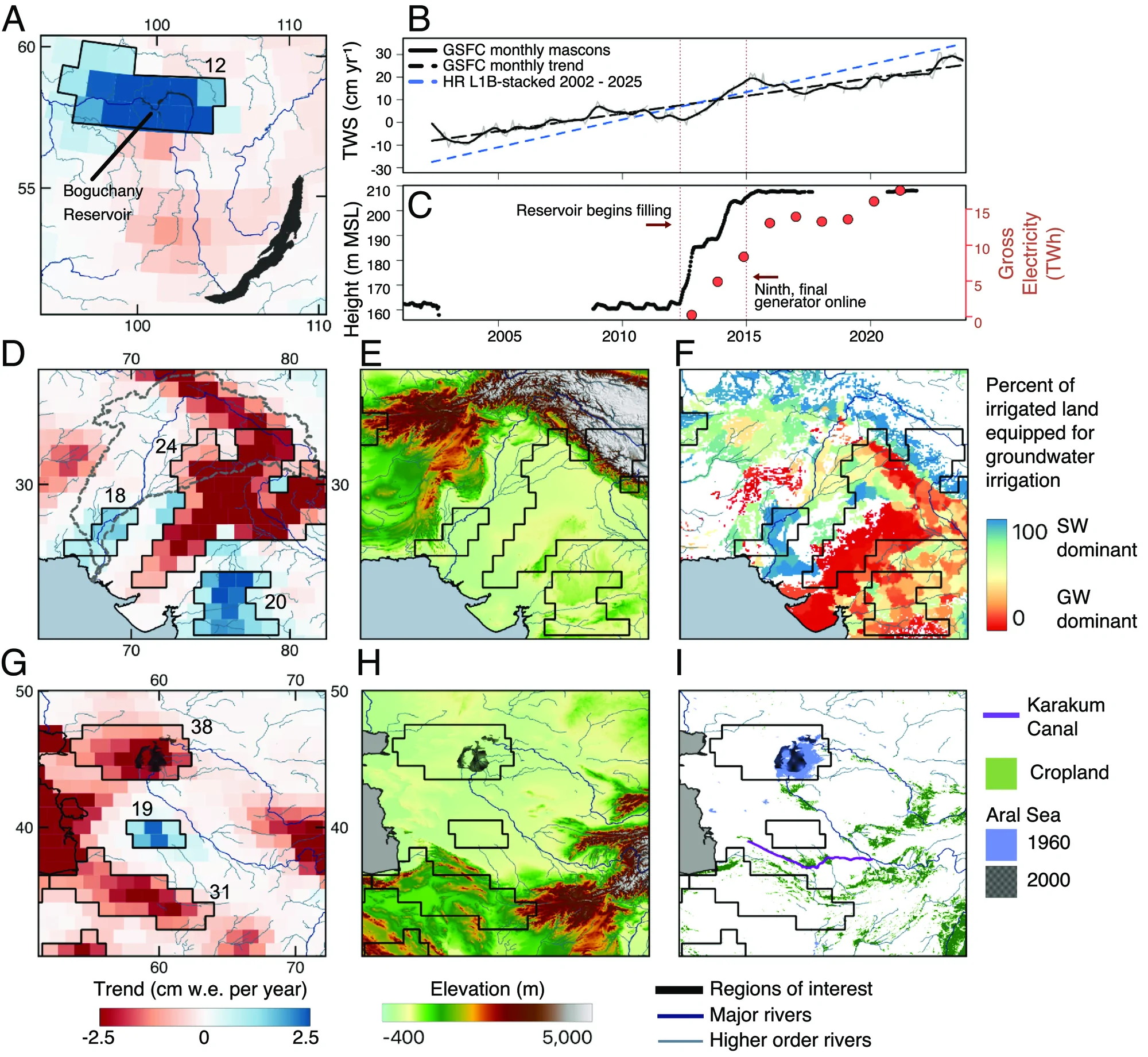
Regions where local dams, diversions, and irrigation runoff impact TWS trends. TWS trends (2002–2025) and human water management surrounding (*A*–*C*) the Boguchany Reservoir, Russia (Region 12), (*D*–*F*) the Aral Sea and Karakum Desert (Regions 36 and 11), and (*G*–*I*) the lower Indus River (Region 10). (*A*, *D*, and *G*) Mapped TWS trends during 2002–2025 (Dataset S1). (*B*) TWS timeseries from monthly mascon solutions (15); slopes of lines represent trends from monthly mascons (black), and from this study’s high-resolution regression evaluated during GRACE/FO (blue). (*C*) Boguchany Dam power production (red) (70) and lake altimetry (black) (71). (*E* and *H*) Elevation above mean sea level. (*F*) Percentage of irrigated land that is equipped for groundwater irrigation over the lower Indus River basin (31). (*I*) Cropland in the Aral Sea Basin (28).

The 3.8 ± 0.9 Gt y^−1^ rate of gain in Region 12 is almost entirely attributable to the 2012–2015 filling of the Boguchany Reservoir (Fig. 4 *G*–*I*), part of Russia’s hydroelectric power cascade on the Angara River. Despite being the largest reservoir by volume built in the 21st century prior to the GERD (33), the Boguchany Reservoir has received little attention in the literature. We estimate that 89.6 ± 22.1 Gt of TWS was gained during the observation period, which is close to the reported reservoir capacity of 58.2 Gt (69) and similar to a high-resolution unregularized GRACE/FO estimate (25). The Boguchany Reservoir is the strongest TWS trend signal in nonpolar northern Asia and explains the majority of variance in our spatial lag model over that continent (Fig. 3*D*).

Malaysia’s Bakun Reservoir is the third largest by volume filled this century, with a storage capacity of 43.8 Gt (33). Together with the upstream Murum Dam, these hydroelectric reservoirs can store up to 55.3 Gt of water within Region 13 (34), where we observed a TWS trend of 2.5 ± 0.5 Gt y^−1^, or 49.8 ± 12.8 Gt total gain during 2002–2025. Incomplete filling and/or ocean signal leakage may account for the discrepancy between the reservoir capacity and the observed gain. With surface areas of approximately 700 km^2^, Bakun reservoir represents one of the smallest hydrologic features that GRACE/FO has measured independently.

The Central Yangtze River Basin (Region 14) gained 3.4 ± 1.1 Gt y^−1^ of TWS, or 79.8 ± 26.4 Gt total, despite lower than average precipitation during the observation period (*SI Appendix*, Table S4). 29 dams have been completed in this region since 2000, with a combined capacity of 77.3 Gt (34). TWS gains were greatest in three mascons directly overlaying the Three Gorges Dam reservoir (*SI Appendix*, Fig. S10), the largest in the Yangtze basin (39.3 Gt) (33) and massive enough to be detected using the standard GRACE/FO technique (1, 66, 72–74).

The Julius Nyerere Dam in Eastern Tanzania (34 Gt capacity) (75) likely contributes to TWS gains in Region 15 (2.2 ± 0.7 Gt y^−1^, or 51.3 ± 16.0 Gt total) despite a lack of a local spatial indicator there (Fig. 3*I*). Conversely, while the 2016 completion of the Gibe III dam in Ethiopia’s Omo River Valley likely contributed to the gains in Region 16, and local spatial associations are present, the reservoir at capacity, 14.7 Gt (33) (*SI Appendix*, Table S3), explains only 13% of the observed TWS trend (4.9 ± 1.0 Gt y^−1^). These regions are likely influenced by positive trends in precipitation around the Rift Valley region (*SI Appendix*, Table S4). Finally, TWS gains in Southeastern Russia (Region 17, 1.9 ± 1.0 Gt y^−1^) were also significantly associated with 21st century reservoir storage. Notably, the Bureya reservoir, filled in 2009, accounts for up to 20.9 Gt of this change (34).

## Varying Effects of Surface Water Irrigated Cropland

At the continental scale, surface water irrigation was a significant predictor of TWS trend spatial patterns in North America and the MENA region (Fig. 3 *A* and *C*), where it exhibited a positive correlation. North America lacked a corresponding LISA cluster, but two important, local positive associations exist in the deserts of Pakistan and Tajikistan (Fig. 3*I*).

The lower Indus River (Region 18), where surface water supplies nearly 80% of local irrigation water (30), has experienced rising TWS (1.0 ± 0.5 Gt y^−1^; Fig. 4 *D–F*). Extreme precipitation in August 2022, brought on by atmospheric rivers and resulting in devastating floods in Pakistan (76), likely contributed to the TWS gains. However, even prior to these events, it was well established that irrigation-enhanced recharge had raised groundwater levels in this region (77). This has not previously been detected and quantified using GRACE/FO, unlike the upper Indus Basin, one of the world’s highest-density groundwater-irrigation areas, where aquifer levels and TWS have been declining (78) (see the groundwater depletion section below for details).

Region 19, in the heart of the Karakum Desert south of the Aral Sea (Fig. 4 *G–I*), experienced a TWS increase of 1.2 ± 0.5 Gt y^−1^ during the study period. TWS gains were concentrated along an ambitious 720-km-long canal project called the Great Turkmen Collector, designed to capture irrigation runoff and transport it to the Karashor Depression (79). Much of that water arrives via the Karakum Canal, the second longest canal in the world, which diverts Amu Darya River water to support desert agriculture to the west (80) (Fig. 4*I*). Because this canal and its branches are largely unlined and flood irrigation is dominant, much of the diverted water evaporates or infiltrates before reaching farmland (81). Other positive local associations exist in Regions 6, 14, and 17, as well as Madhya Pradesh (Region 20, 3.5 ± 0.8 Gt y^−1^) and Southeastern India (Region 21, 1.0 ± 0.5 Gt y^−1^). Although these last two were significant, the more likely driver was precipitation, which was above average during the observation period and has increased at a rate of 4-5 mm y^−1^ since 1979 (*SI Appendix*, Table S4).

However, surface water irrigated area is strongly collinear with groundwater-irrigated area in several continents (*SI Appendix*, Fig. S9). Although VIF values remain below conventional multicollinearity thresholds (*SI Appendix*, Table S7), the spatial autoregressive model likely cannot fully isolate the independent contributions of surface and groundwater irrigation where the two co-occur extensively. Inclusion of an interaction term between the two irrigation types yielded negligible improvement in model fit and did not meaningfully alter continental-scale coefficient estimates. Negative local associations between TWS trends and surface water irrigated area are consequently concentrated in regions where groundwater irrigation is also present (Fig. 3).

## Groundwater Depletion Is More Intense than Previously Estimated

**Fig. 5.**
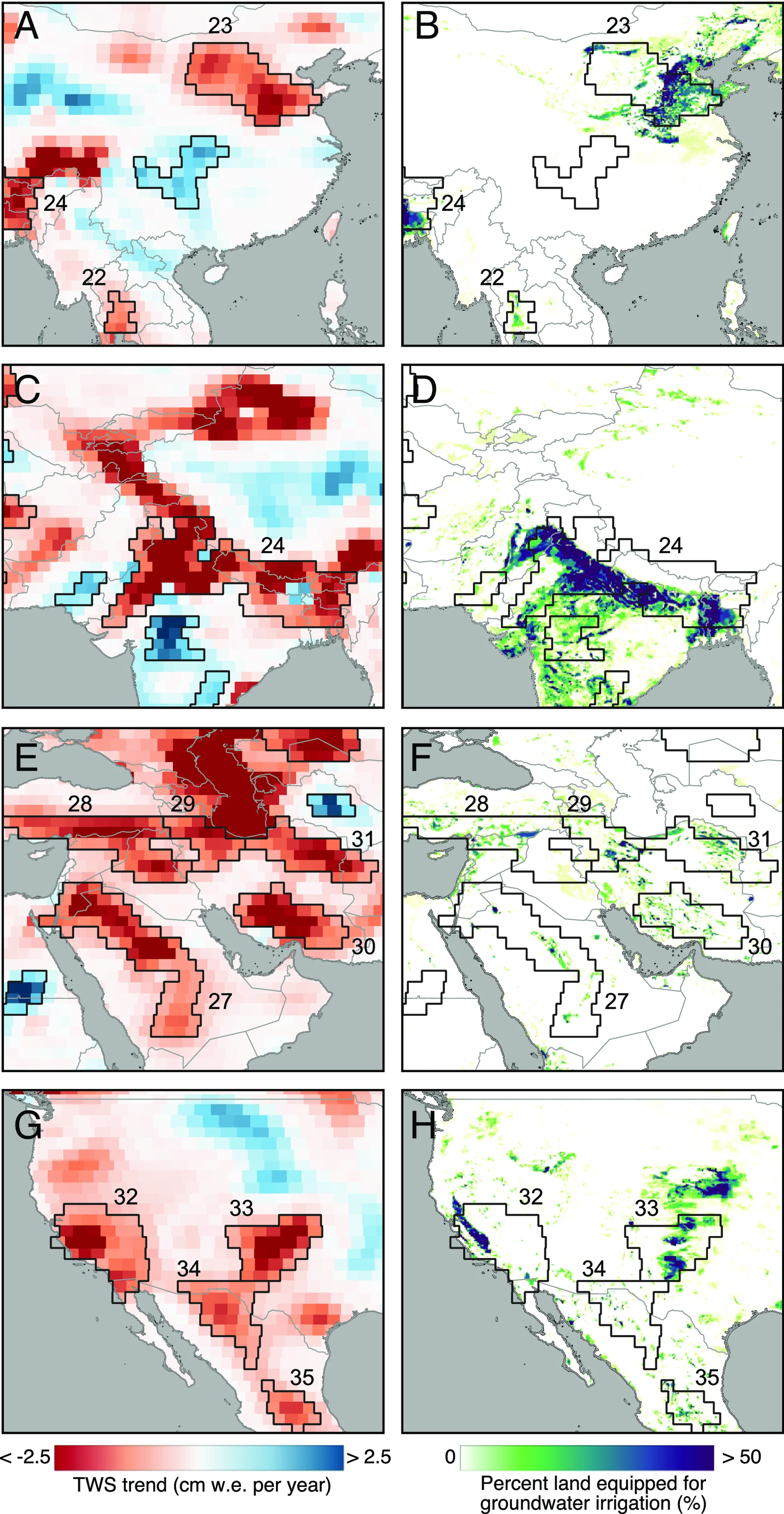
High spatial agreement between TWS losses and groundwater-irrigated cropland. TWS trends during the GRACE and GRACE-FO period (*Left*, Dataset S1) and percent of land equipped for groundwater irrigation (31) (*Right*) over (*A* and *B*) eastern Asia (Regions 17, 22, and 23), (**C* and *D**) southern and high mountain Asia (Regions 20, 21, and 24), (**E* and *F**) the Middle East (Regions 26 to 30), and (**G* and *H**) North America (Regions 31 to 34).

Since GRACE first revealed severe, agriculture-driven groundwater depletion in northern India (82, 83), gravimetry has been applied in hundreds of aquifer studies (10), including in Asia (Regions 22 to 24), the Middle East and North Africa (Regions 25 to 31), and the Americas (Regions 32 to 36). Groundwater-irrigated cropland area was a significant spatial predictor of TWS trends for spatial lag models in half of the continental regions (Fig. 3). New maps of declining TWS more closely resembled patterns of irrigation than those from previous approaches (Fig. 5 and *SI Appendix*, Fig. S6), and our results show that TWS losses associated with agricultural irrigation are considerably more acute than those measured by monthly mascon products (Fig. 2 and *SI Appendix*, Table S6) or previously reported in literature (*SI Appendix*, Table S8). Although the dearth of global in situ groundwater storage observations precludes formal product validation, comparison of aquifer-level well depth trends (84) against L1B-stacked TWS trends indicates that our product yields steeper regression slopes and stronger correlations with observed water level decline than the benchmark GSFC monthly mascon trends (*SI Appendix*, Fig. S11).

The important variables influencing TWS trends in southern Asia were glacier mass loss, reservoir impoundment, precipitation trend, and groundwater-irrigated land area (Fig. 3*G*). Beyond the direct effects of aquifer pumping, declining precipitation likely reduces surface water availability, driving increased reliance on groundwater resources to compensate for surface water deficits.

Our estimated trend in the extensively irrigated Chao Phraya River Basin of Thailand (Region 22, −1.0 ± −0.5 Gt y^−1^) is similar to that from another recent study (85), despite elevated precipitation following their study period (2002–2020), which one would expect to dilute the trend. Due to the agricultural implications (*SI Appendix*, Table S3), groundwater losses in the North China Plain (Region 23) and Northern India (Region 24) have been heavily scrutinized. For the former, we computed a trend of −7.9 ± 2.0 Gt y^−1^, which is similar to recent estimates of −6 to −8 Gt y^−1^ (86, 87) when adjusted for spatial area. However, for Northern India, the trend (−32.4 ± 4.0 Gt y^−1^, or −27.5 Gt y^−1^ when glacial mass loss is removed) is considerably higher than other recent estimates [−15.3 Gt y^−1^ (88), −18.1 Gt y^−1^ (89), scaled for area differences]. Here, slightly above average precipitation during the GRACE/FO period (*SI Appendix*, Table S4) could not overcome groundwater exploitation. Himalayan glacier retreat [−4.8 Gt y^−1^ (26)] explains only 15% of the TWS change, consistent with earlier findings (82).

We inferred an overall depletion rate in the MENA region of −42.0 ± 7.7 Gt y^−1^, similar to another recent estimate (90) but focused within less than 3 million km^2^ (Regions 25 to 30) as opposed to 7 million km^2^ in the previous study. MENA’s vast, fossil (91) aquifers are increasingly stressed by desert agriculture (30) and a warming climate (92). Continental spatial lag models identified groundwater-irrigated land area as the strongest predictor of TWS trend spatial variance in the Sahara and Middle Eastern Deserts (Fig. 3*C*).

In the North-Western Sahara Aquifer System (Region 25, NWSAS), one of the world’s largest transboundary aquifers, groundwater withdrawals (2.75 Gt y^−1^) greatly exceed recharge (1.3 Gt y^−1^), resulting in dewatering of artesian wells and natural springs (91). Region 25 encompasses the northern terminus of the NWSAS, the Chotts basin, where piezometric heads are highest but are rapidly declining due to a combination of groundwater withdrawals and a significant downward trend in annual precipitation (*SI Appendix*, Table S4) (91–93). Previous studies reported 3.0 to 3.7 Gt y^−1^ of TWS loss over one million km^2^ of the NWSAS (*SI Appendix*, Table S8) (36, 93, 94). We estimated that 4.5 ± 0.9 Gt y^−1^ was lost from an area of just 310,000 km^2^. Northern, coastal Morocco (Region 26) lost −1.2 ± 0.6 Gt y^−1^, approximately three times higher than another GRACE-based estimate from 2022 (95).

In Regions 27 to 31, 35 to 91% of irrigated cropland is equipped for groundwater extraction (31), and spatial patterns of TWS depletion follow those of irrigation (Fig. 5). Groundwater overexploitation, climate change, and a historically severe drought caused a total loss of 851 Gt during the study period. In Region 27 alone, TWS losses were 12.7 ± 1.7 Gt y^−1^ [41% more than a recent estimate (96)], over six times Saudi Arabia’s annual renewable water supply (97) and largely affected the Saq aquifer (98). In Türkiye [Region 28, −8.6 ± 1.5 Gt y^−1^, over double a recent estimate (99)], an increase in the country’s cropland productivity, combined with the 2007–2009 severe Fertile Crescent drought, disturbed important downstream water resources for Syria and Iraq. The resulting water shortages likely exacerbated Syria’s socioeconomic and political tension during Arab Spring (100, 101). Region 29 (−2.9 ± 0.8 Gt y^−1^) encompasses heavily irrigated lands in northwestern Iran. Signal leakage from the Caspian Sea complicates estimation of adjacent trends and may explain why the loss rate computed by monthly mascons exceeded our estimate for Region 29, unlike most other regions (Fig. 2 and *SI Appendix*, Table S6). Maximum TWS depletion in Iran occurred in the Salt Lake basin (Region 30, −6.8 ± 1.2 Gt y^−1^), which supplies water to over a quarter of the country’s population (102) and where groundwater-irrigated pistachio cultivation has caused rapid water table declines (103). The Central Desert to the northeast (Region 31) has lost 5.3 ± 1.2 Gt y^−1^. These TWS declines were poorly resolved in previous GRACE/FO based studies (*SI Appendix*, Table S8). One reported that Iran was losing 12 Gt y^−1^ overall (104), while our results show 12.1 Gt y^−1^ lost from just one quarter the nation’s area (Regions 30 and 31).

Aquifer depletion is likewise an issue in agriculturally productive regions of the Americas. Record-breaking temperatures and a series of droughts, consistent with climate change predictions, have plagued the Southwestern U.S. (Region 32) since 2003, including the worst single year (2014) moisture deficit in the last millennium (38). Exacerbated by increased reliance on groundwater (105), TWS has declined by 6.8 ± 1.3 Gt y^−1^, centered on California’s Central Valley (Fig. 5). Droughts also contributed to depletion of the southern High Plains Aquifer (Region 33, −5.9 ± 1.3 Gt y^−1^), where more than 80% of irrigated land relies on groundwater (31).

In Mexico, droughts have forced municipalities and farms to rely increasingly on groundwater, causing well-documented land subsidence (106). Region 34 (Chihuahua, −3.8 ± 1.1 Gt y^−1^) is home to the most overexploited aquifers in Mexico (107), with groundwater extraction rates rapidly increasing (108). Nevertheless, the region is understudied because only 6% of the land is classified as irrigated (109) (*SI Appendix*, Table S3) and monthly mascons underestimate the trend (*SI Appendix*, Table S6). Region 35 (central Mexico, surrounding Mexico City) experienced a TWS decline of 1.8 ± 0.6 Gt y^−1^, which was underestimated in previous literature (*SI Appendix*, Table S8) (88). In the Brazilian Highlands (Region 36), low precipitation (*SI Appendix*, Table S4) during two consecutive strong El Niño periods (2015–2017 and 2019–2020) (92), paired with groundwater-fed irrigation (*SI Appendix*, Table S3) produced a TWS decrease of 24.3 ± 2.5 Gt y^−1^. As with most other pumping-impacted regions, a significantly positive trend in vapor pressure deficit likely exacerbated the deficit (*SI Appendix*, Table S5).

## Diversions, Regulations, and Nonlocal Anthropogenic Impacts

An important limitation to the statistical spatial association methods used here is the dependence on local relationships between human activities and TWS trends. Nonlocal activities, such as upstream damming, diversions or bifurcations of rivers, connectivity between exploited and unexploited aquifers, and land–atmosphere feedbacks, were not reported but can be paramount. Less than one quarter of the world’s long, exorheic rivers remain unmanaged (110), and endorheic lakes have seen widespread decline over the last century (37, 111). Several notable TWS trends are most certainly caused by nonlocal human activities despite not being identified by our statistical methods. Although the type of spatial model we used here (a Durbin model) (112) accounts for spatial spillovers in both the dependent and independent variables, which would in theory resolve nonlocal relationships, we found that the significant autocorrelation in the GRACE data (even after high-resolution L1B stacking) produced large autocorrelation terms and a dense spatial weights matrix that rendered the separation of local and nonlocal influences unstable. We therefore address several well-documented hotspots through direct discussion rather than statistical attribution.

Region 37 (0.9 ± 0.5 Gt y^–1^) directly overlays Phase II pumping fields from Libya’s Great Manmade River Project. The depletion here is likely attributable to the project’s intensive pumping, which at capacity would route approximately 2.2 Gt y^–1^ to heavily irrigated regions along Libya’s northern coast; however, operations have not reached capacity since the collapse of the Gaddafi regime in 2011 (36).

Another well-known example is the Aral Sea (Region 38, −4.9 ± 1.1 Gt y^−1^), which has shrunk by 74% from its mid-20th century surface area of 66,000 km^2^ (111) due to extensive diversions of and withdrawals from its contributing rivers, in particular the rerouting of the Amu Darya River flows to the Karakum canal. While the sea’s desiccation is well known, the relative contributions of climate change and anthropogenic factors are not (113). We found that the rate of TWS gain in Region 11 (the Karakum desert) was equivalent to about one fourth of the loss rate of the Aral Sea.

A similar story, on a smaller scale, may be told for Mar Chiquita (Region 39, −1.6 ± 0.5 Gt y^−1^), an endorheic saline lake in Argentina that is essential to flamingo breeding populations and a local tourist industry (114). Droughts, climate change, and diversions for agriculture have led to its desiccation (115). Currently holding around 14.5 Gt of water (116), the lake could transition to a salt flat within a few decades should this trend continue.

Management decisions affecting established dam operations can substantially influence water impoundment and downstream resource availability yet remain difficult to monitor remotely. We attribute an intense positive TWS trend in southern Egypt (Region 40, 2.3 ± 0.6 Gt y^–1^) to recent increases in the volume of Lake Nasser, to which a small but significant positive precipitation trend also contributed (*SI Appendix*, Table S4). In anticipation of reduced Nile inflow following construction of the Grand Ethiopian Renaissance Dam upstream, Egypt has prioritized reservoir retention while aggressively releasing or pumping excess Nasser storage to the adjacent Toshka depressions. Assigning the 2022 Toshka Lakes volume [58 Gt; (117)] to the corresponding mascon’s reservoir storage covariate yielded a significant positive association between reservoir impoundment and TWS trend across the MENA region, with a slight improvement in overall model fit (*SI Appendix*, Fig. S12).

## Implications

The 40 regions discussed here represent the strongest examples of direct human influence on 21st-century TWS trends, but anthropogenic impacts on TWS are by no means limited to these hotspots. Nonlocal feedbacks, scale disparities, unreported land and water use, collocated natural trends, and outdated datasets may obscure additional consequences for water resources. Notably, regions without significant TWS trends can be equally informative. For instance, we found a strong local positive association between TWS trends and rainfed cropland area in the Pampas region (Fig. 3*I*). Here, TWS trends are relatively neutral (Fig. 1), despite widespread drought and negative TWS trends in surrounding regions. This led to the significant positive local relationship, likely influenced by the continental-scale dependence on rainfed cropland area in the multivariate regression model, and corresponding to observed groundwater rising as crops replaced native vegetation (118).

A key conclusion from this study is that freshwater storage trends in many heavily managed basins and aquifers are more acute than previously estimated, which has implications for food and water security, hydrological and climate modeling, and comprehension of the delicate balance of Earth system processes. Previously published estimates of TWS declines associated with groundwater pumping were on average 45% lower than ours, even when accounting for differences in spatial and temporal domains (*SI Appendix*, Tables S6 and S8). For example, in Central Saudi Arabia (Region 27), the difference between TWS trends from the conventional and high-resolution approaches exceeds that nation’s internal renewable water resources [2.5 Gt y^−1^, (119)] by nearly a factor of two. Further, TWS gains associated with land use and change in equatorial zones have consequences for downstream water resources, especially when land–atmosphere interactions and teleconnections are considered (120). High-resolution TWS trend measurements around large lakes and rivers complement altimetry observations to better quantify seepage from reservoirs (121) and irrigation enhanced recharge.

Applying this regression approach, GRACE/FO can detect TWS trends with far greater spatial detail and without the need for auxiliary data or downscaling methods. The increased resolution makes satellite gravimetry useful for tracking freshwater resources in more poorly monitored regions where few alternatives exist. However, by integrating across the full observational record, the Level-1B regression precludes the retrieval of temporal dynamics including interannual variability, and transient hydrological responses to climate forcing. Comprehensive water resource assessment for individual basins may benefit from pairing the spatially refined secular trends with traditional GRACE and GRACE-FO monthly mascon time series to recover the full spatiotemporal character of TWS change.

The successor to GRACE-FO, GRACE Continuity (122) is expected to launch in 2028, and the European Space Agency intends to launch a Next Generation Gravity Mission a few years later (123). As the data record grows, this approach can be adjusted to further enhance the spatial resolution of the long-term trends.

## Materials and Methods

### Gravity-Derived TWS Trends.

In this study, TWS trends during April 2002 to November 2025 were derived using GRACE/FO Level-1B (L1B) intersatellite range rate observations. Traditional approaches for finding TWS trends utilize the standard Level 2 time-variable gravity product, provided as a set of monthly, unregularized spherical harmonic coefficients (124), or, more commonly, the Level 3 global mass concentration (mascon) solutions (14, 125), which bypass much of the filtering necessary to mitigate signal attenuation in the Level 2 products (126). TWS trends are typically found by linear regression of the deseasoned monthly anomalies (14).; thus, trend resolution is limited to that of the monthly Level-2 and Level-3 products themselves, or at best 100,000 to 150,000 km^2^ at mid-latitudes (15). However, since early in the mission, static gravity fields, which show Earth’s long-term mean distribution of mass, were derived from GRACE at much higher spatial resolution than a single month of data could achieve, by optimizing the retrieval of fine scale spatial information at the expense of temporal information. Our TWS trend solution leverages this trade-off between temporal and spatial resolution by fitting a regression model directly from the L1B data for each of the mascons (i.e., “stacking” the L1B data). The concept of coestimating other components along with the mean is not new; for example, the GOCO (127) and EIGEN SH (128) models calculate time-variable fields (trend and annual signal). However, NASA Goddard Space Flight Center is the first to extend the approach to regularized mascon estimation.

The regularized L1B regression mascon solution coestimates trend, bias, and annual sinusoid terms at 41,168 1-arc-degree equal area mascons from more than 23 y (April 2002 to August 2016 and June 2018 to November 2025) of L1B data. Approximately the last year of the GRACE mission (through June 2017) was not included in our analysis given the loss of accelerometer functionality after August 2016 (22). The L1B regression improves spatial resolution, resolving trends at approximately 12,500 km^2^, and has been applied to TWS trends in High Mountain Asia (23), global ice sheet analysis (22), and climate driven TWS losses in the Great Basin, United States (24). The L1B data processing utilized state-of-the-art background models, estimation procedures, and glacial isostatic adjustment (GIA) and seismic corrections.

The L1B regression applied in this study is nearly identical to that used in these recent studies (22–24); however, in addition to extending the observation timeframe, we applied two improvements to the regularization, including a latitude-dependent polar sampling bias correction, and a resolution adjustment to the a priori background model. Our TWS trend map is shown in Fig. 1 and is available in Dataset S1 (DataS1_TWS_Trend_200204_202511.txt).

Uncertainty for each region is defined as twice (2-*σ*) the root sum square (RSS) of noise and GIA errors. Noise uncertainty was characterized by examining the difference between TWS trend maps from L1B-regression and from the GOCO-06 s spherical harmonic solution. Significant hotspots of TWS trends were delineated by combining groups of at least four mascons exceeding noise uncertainty (*SI Appendix*, Fig. S1). As in (22), we define GIA uncertainty for a region as the spread (1-*σ*) of mean mascon corrections from three GIA models (129–131). The reported uncertainties for the regions highlighted in this study average ±28% of trend magnitude and are comparable to or smaller than the interproduct spread among standard monthly mascon solutions (*SI Appendix*, Table S2), indicating that this product carries similar uncertainty and water balance closure limitations as those encountered with conventional GRACE-based assessments.

We compared the L1B-Stacked solution to TWS trends derived from monthly mascon products from NASA Jet Propulsion Laboratory (14), and from the University of Texas Center for Space Research (16), using least squares regression of regularized monthly timeseries (including linear trend, sinusoidal seasonality, and error terms). *SI Appendix*, Table S1 provides a summary of the key attributes of all GRACE-related products, the regression technique and timeframe used for each, and a brief description of how they were applied in this study.

### Land and Water Use, Climate, and Glacial Mass Loss Data.

We evaluated the spatial association and co-occurrence of extremes between global TWS trends and spatially distributed land and water use datasets. Anthropogenic variables used in the statistical analysis included percent rainfed cropland area, percent groundwater-irrigated land area, percent surface water-irrigated land area, percent deforested land area, and summed volumetric reservoir storage capacity from dams built in the 21st century.

Rainfed cropland area was provided by high-resolution map of global rain-fed, irrigated, and paddy croplands (GRIPC) (29). To estimate the percent of land that was equipped for irrigation, as well as groundwater or surface-water equipped irrigation fractions, we used the FAO Global Map of Irrigated Areas (GMIA, version 5) (31). The product of these two values was the percent of land area equipped for surface water or groundwater irrigation. To estimate deforested area within each region, we used the change in forested area from 1960 to 2019 from the Historic Land Dynamics Assessment + (HILDA+) dataset (28). Storage capacity information for dams and global reservoirs was retrieved from the Global Reservoir and Dam Database (GRanD, v1.3). (34). GRanD v1.3 includes 7,320 large (at least 0.1 km^3^ reservoir capacity), georeferenced dams, and corresponding reservoir outlines. Three large reservoirs filled since the most recent version of GRanD were manually georeferenced and added to the dataset: the Julius Nyerere reservoir in Tanzania (75), the Baihetan reservoir in China (132), and the Grand Ethiopian Renaissance Dam (68). To find the region-aggregate total dam storage filled during the 21st century, we identify all dams completed after 2000 within a given mascon or region of interest and find the sum (in Gt) of the representative maximum storage capacity. The volumetric storage of reservoirs overlapping multiple mascons was distributed across those mascons proportional to the overlapping surface area percentage. Regional statistics and global patterns of land use can be found in *SI Appendix*, Fig. S3 and Table S3, respectively.

For Fig. 4, we obtained reported gross electricity production data from the Boguchany hydropower plant between 2012 and 2020 from RusHydro (70). We also obtained satellite radar altimetry data from the Global Reservoirs and Lakes Monitor (G-REALM) (71) for the Boguchany reservoir.

We attributed TWS trends in part to natural climate variability based on trends and anomalies in data obtained from the ERA5 fifth generation ECMWF reanalysis (27), aggregated to mascon resolution using area weighting. The GRACE/FO period precipitation deviation (expressed as percent) from long term (40 y) mean was found by taking the ratio of mean 2002–2025 annual precipitation and the mean 1981–2025 annual precipitation. We calculated trends in the ERA5 annual precipitation timeseries for the 2002–2025 (GRACE/FO) period using Sen’s nonparametric estimator of slope (133). Trends were evaluated for significance (*P* < 0.05) with the Mann–Kendall test for monotonic trend (134). ERA5 precipitation anomalies, trends, and their significance are shown at mascon resolution in *SI Appendix*, Fig. S4. Region-aggregate precipitation trends and anomalies are provided in *SI Appendix*, Table S4. We repeated the analysis of trends, long-term mean, and GRACE/FO-period anomalies on ERA5 global vapor pressure deficit, with results shown in *SI Appendix*, Fig. S4 and Table S5.

For Northern India (Region 22), loss of glacial mass storage due to global warming contributes (at least in part) to storage losses. To estimate the contribution of glacial mass change to the total TWS trend, we aggregated a recent dataset of global glacier mass loss observations from Hugonnet et al. (26) at mascon resolution with area weighting.

### Statistical Associations Between TWS Trends and Anthropogenic, Climate Variables.

To evaluate the significance of spatial associations between global land TWS trends and anthropogenic or climate variables, we used a sequential analytical framework combining spatial Durbin models (SDM) (112) and bivariate local indicators of spatial association (LISA) (47) using Lee’s L statistic (41) (*SI Appendix*, Fig. S13). In both methods, accounting for spatial autocorrelation is critical because datasets with high spatial dependence or smoothing (such as GRACE) violate the assumption of independent observations, leading to biased inference, incorrect significance testing, and misinterpretation of underlying spatial processes.

We first characterized the autocorrelation structure of TWS trends by fitting a power-law function to the distance-decay of Moran’s I (135) (a measure of the degree to which nearby locations have similar values) across spatial lags, identifying the neighborhood bandwidth at which spatial autocorrelation becomes negligible. This decay function, defining the spatial weights matrix used in subsequent models, varied by continent (*SI Appendix*, Fig. S14). SDM results were generally rigorous to slight changes in spatial weights characterization, as long as this decay function was well specified (*SI Appendix*, Fig. S15).

We then applied continental-scale SDMs with mascon TWS trends as the dependent variable and mascon-aggregated anthropogenic and climate variables as predictors. SDM is an extension of the spatial lag model, a regression framework that incorporates the spatially weighted average of neighboring response values as a predictor, allowing outcomes at each location to depend explicitly on outcomes at neighboring locations (136). SDM additionally incorporates spatially lagged independent variables, capturing how the spatial distribution of predictors in neighboring locations influences the local response (for instance, local groundwater pumping may have consequences downstream or within the greater aquifer system). This requires each driver to account for variance in TWS trends in competition with all other predictors and the broad-scale autocorrelation structure, providing a rigorous basis for inferring potential causality.

Independent variables were divided into mascon-scale anthropogenic predictors (percent rainfed cropland area, percent groundwater-irrigated land area, percent surface water-irrigated land area, percent deforested area, and area-weighted sum of 21st century impounded reservoir storage) and climate predictors (trends and anomalies in precipitation and vapor pressure deficit during the GRACE/FO period, and glacial mass loss).

Predictors identified as significant in the continental-scale SDM were then tested using bivariate LISA, a family of local statistics that identify clusters where spatial correlations between two variables are significantly stronger or weaker than expected by chance (47). We used Lee’s *L* statistic, a normalized index of spatial codistribution between two variables (41). Unlike common correlation metrics such as Pearson’s *r*, Lee’s *L* penalizes correlations between highly autocorrelated variables, preventing significance inflation. Regions of significant TWS trend hotspots were considered to be associated with anthropogenic factors if SDM results indicated a significant spatial association with one of the anthropogenic independent variables, and LISA identified significant local copatterning between TWS trend and the predictor.

Additional information on these methods may be found in *SI Appendix*.

## Supplementary Material

Appendix 01 (PDF)

Dataset S01 (TXT)

## Data Availability

Previously published data were used for this work (16, 23, 26–30, 33, 39). All other data are included in the manuscript and/or *SI Appendix*.
