# Supplementary material for: Spatially refined satellite gravimetry captures human signatures in global terrestrial water storage trends
Source: Proc Natl Acad Sci U S A. 2026 Sep 14;123(38):e2600775123. doi: 10.1073/pnas.2600775123 (PMC13598224; doi:10.1073/pnas.2600775123)
Supplement: Supplementary file 1 [file pnas.2600775123.sapp.pdf]

PNAS

Supporting Information for

Spatially refined satellite gravimetry captures human signatures in global terrestrial water storage trends

Mary Michael O'Neill, Matthew Rodell, Bryant D. Loomis

Corresponding author: Mary Michael O'Neill
Email: mary.m.oneill@nasa.gov

This PDF file includes:

Supporting text
Figures S1 to S15
Tables S1 to S8

Other supporting materials for this manuscript include the following:

Dataset S1

Supporting Information Text

S.1 Level-1B stacking regularization and global TWS trend processing

S.1a: Background geophysical models for GRACE and GRACE/FO processing

We used the microwave interferometer data for both GRACE and GRACE-FO, along with the RL06 AOD1B atmosphere and ocean de-aliasing background geophysical model (1). While most monthly mascon products restore the AOD1B ocean surface pressure (GAD), L1B regression does not, meaning that trends are consistent with the Level 2 GSM (GRACE Satellite-only Model) product. Sun et al. (2) was used to correct trends in Earth's geocenter (degree 1), and C_{20} coefficients (degree 2 order 0 Stokes coefficients, which represent changes in Earth's dynamic oblateness) were replaced using satellite laser ranging (SLR) derived values according to Loomis et al. (3). Given inaccuracies in C_{30} GRACE and GRACE-FO estimates when operating in single accelerometer mode, C_{30} coefficients from GRACE-FO were replaced with SLR values (3). The ICE-6G_D model (4) was used for glacial isostatic adjustment (GIA) correction. We also included a gravity model of the five largest coseismic events during the GRACE period (5).

S.1b: Regularization

In this section, we compare the regularization procedure for L1B time series regression with explicit regression of the GSFC monthly mascons (6). First, let us examine the GSFC least-squares monthly mascon estimate for $\widehat{\mathbf{m}}_j$, the jth month, given by the Tikhonov regularization (7):

$$\widehat{\mathbf{m}}_j = \left(\mathbf{A}_j^{\mathrm{T}}\mathbf{W}_j\mathbf{A}_j + \lambda\mathbf{P}_j\right)^{-1}\mathbf{A}_j^{\mathrm{T}}\mathbf{W}_j\mathbf{d}_j \qquad (1)$$

In Equation 1, the design matrix $\mathbf{A}_j$ relates L1B inter-satellite range data to the mascons, $\mathbf{W}_j$ and $\mathbf{P}_j$ are the inverses of the data noise covariance and the signal noise covariance, respectively, λ is a tunable regularization parameter, and $\mathbf{d}_j$ is the inter-satellite residuals vector (8, 9). Equation 1 assumes static adjustments within the month j, and the time series of kth mascon, $\widehat{\mathbf{m}}_k^{\mathrm{T}} = \left[\widehat{m}_{1,k} \ldots \widehat{m}_{j,k} \ldots \widehat{m}_{N_t,k}\right]$ is constructed from $j = 1, \ldots, N_t$ estimates for all available months. In the explicit regression of GSFC monthly mascon timeseries (8), $\widehat{\mathbf{m}}_k^{\mathrm{T}}$ is fit with a temporal basis function $\mathbf{F}$ with N_f terms, whose multipliers $\hat{\mathbf{x}}_k$ are given by

$$\hat{\mathbf{x}}_k = (\mathbf{F}^{\mathrm{T}}\mathbf{F})^{-1}\mathbf{F}^{\mathrm{T}}\widehat{\mathbf{m}}_k \qquad (2)$$

In this study, basis functions include bias, trend, and annual sinusoidal fit, represented by $F_{j1} = 1, F_{j2} = t_j$, and $F_{j3} = \cos 2\pi t_j$ and $F_{j4} = \sin 2\pi t_j$, respectively. The simultaneous inversion for all global mascons is given by

$$\hat{\mathbf{x}} = [(\mathbf{F}^{\mathrm{T}}\mathbf{F})\mathbf{F}^{\mathrm{T}} \otimes \mathbf{I}]\, \widehat{\mathbf{m}} \qquad (3)$$

In Equation 3, $\hat{\mathbf{x}}^{\mathrm{T}} = \left[\hat{\mathbf{x}}_b{}^{\mathrm{T}}\hat{\mathbf{x}}_t{}^{\mathrm{T}}\hat{\mathbf{x}}_c{}^{\mathrm{T}}\hat{\mathbf{x}}_s{}^{\mathrm{T}}\right]$ represents the inversion of bias, trend, sine, and cosine multipliers gathered into vectors ($\hat{\mathbf{x}}_b, \hat{\mathbf{x}}_t, \hat{\mathbf{x}}_c$, and $\hat{\mathbf{x}}_s$, respectively) of length $N_S = 41{,}168$ mascons (8). The collection of timeseries for all N_s mascons is $\widehat{\mathbf{m}}^{\mathrm{T}} = \left[\widehat{m}_1 \ldots \widehat{m}_j \ldots \widehat{m}_{N_t}\right]$, and $\mathbf{I}$ is an identity matrix.

Thus, to explicitly find the trend component $\hat{\mathbf{x}}_t$ from GSFC monthly mascons in Equation 2, we must first solve for monthly mascon timeseries, $\widehat{\mathbf{m}}_j$ in Equation 1, which is then fit with defined basis functions. The L1B regression method, instead, solves for $\hat{\mathbf{x}}$ directly from $\mathbf{d}_j$ in Equation 1 with

$$\hat{\mathbf{x}} = [(\mathbf{F}^{\mathrm{T}} \otimes \mathbf{I})(\mathbf{A}^{\mathrm{T}}\mathbf{W}\mathbf{A} + \mathbf{P})(\mathbf{F} \otimes \mathbf{I})]^{-1}(\mathbf{F}^{\mathrm{T}} \otimes \mathbf{I})\, \mathbf{A}^{\mathrm{T}}\mathbf{W}\mathbf{d} \qquad (4)$$

where the $\mathbf{A}_j$ design matrix, $\mathbf{W}_j$ noise covariance and $\mathbf{P}_j$ signal covariance from Equation 1 are the jth blocks in the block-diagonal matrices $\mathbf{A}$, $\mathbf{W}$, and $\mathbf{P}$, respectively, and $\mathbf{d}^{\mathrm{T}} = \left[\mathbf{d}_1^{\mathrm{T}} \ldots \mathbf{d}_{\mathrm{j}}^{\mathrm{T}} \ldots \mathbf{d}_{\mathrm{N_t}}^{\mathrm{T}}\right]$ are the inter-satellite observations (8). However, instead of the block-diagonal structure used for the regularization matrix in the monthly mascons, the L1B regression utilizes a diagonal regularization matrix, such that the term $(\mathbf{F}^{\mathrm{T}} \otimes \mathbf{I})\mathbf{P}(\mathbf{F} \otimes \mathbf{I})$, corresponding to signal covariance, neglects cross-covariance and contains only auto-covariant terms in the multipliers (6, 8). The reduced strength of the simple diagonal regularization enforces less artificial structure and facilitates the high resolution afforded by the L1B regression technique, relative to the explicit monthly mascon method. Diagonal terms in the regularization correspond to the monthly mascon signal variance of particular $\hat{\mathbf{x}}$ components; for example, the trend diagonal term is given by $1/\sigma_{t_j}^2$, where $\sigma_{t_j}^2$ is the signal variance of the trend component from the explicit GSFC monthly mascon regression (6).

The parameter λ, which defines the mascon signal covariance matrix $\lambda^{-1}\mathbf{P}^{-1}$, may dampen or strengthen the degree of regularization and was determined by minimizing the differences between the L1B regression trend component and GOCO-06s solution (6).

The above regularization was demonstrated in Loomis et al. 2019, Loomis et al., 2021, and Hall et al., 2024 (6, 9, 10). We have updated the regularization to include modern GRACE-FO years, and we made two important improvements since these publications. First, we implement a modification to the noise covariance weighting scheme to account for the latitude-dependence in data coverage by scaling weights by $\frac{1}{\sqrt{n}}$, where n is the number of observations for a given latitude band. This was successful in reducing noise in high latitude ocean and ice sheet mascons, but it had little effect on most land mascons.

The second modification addresses a mismatch in spatial resolution between the lower-resolution time-variable background gravity field and the high-resolution estimate. To improve the orbits of the GRACE satellites, we include a time-variable gravity background field to degree/order 60 when processing the L1B data. In earlier Level-1B stacking applications (6, 9, 10), the total trend estimate was equal to the sum of the background field and the "Delta" high-resolution estimate. We now eliminate any mismatch in spatial resolution between these fields by modifying the right-hand-side of the normal equation so that the a priori mascon values are reset to zero prior to inverting:

$$\mathrm{RHS}_{mod} = \mathrm{RHS}_{orig} + \mathbf{A}^{\mathrm{T}}\mathbf{A}\left(\mathbf{m}_{bkg}\right) = \mathbf{A}^{\mathrm{T}}\mathbf{d} + \mathbf{A}^{\mathrm{T}}\mathbf{A}\left(\mathbf{m}_{bkg}\right), \quad (5)$$

where $\mathbf{A}$ is the design matrix containing the partial derivatives of the measurements relative to the mascons, $\mathbf{d}$ is the inter-satellite residuals vector, and $\mathbf{m}_{bkg}$ is the background model. This produces a high-resolution trend estimate that is not "corrupted" by the lower resolution background model.

S.1c: TWS trend map spatial resolution

Mathematically, the inherent resolution of the regularized mascon solution is best understood by examining the bias (9), which is given by

$$\mathbb{E}[\hat{\mathbf{x}} - \mathbf{x}] = (\mathbf{R} - \mathbf{I})\mathbf{x} \quad (6)$$

where $\hat{\mathbf{x}}$ is the final mascon $N_f \times N_s$ estimate solution matrix (or in the case of monthly mascons, $N_t \times N_s$), $\mathbf{x}$ is the truth state, and $\mathbf{R}$ is the resolution operator given by

$$\mathbf{R} = (\mathbf{A}^{\mathrm{T}}\mathbf{W}\mathbf{A} + \mathbf{P})^{-1}\mathbf{A}^{\mathrm{T}}\mathbf{W}\mathbf{A} \quad (7)$$

If noise is neglected, this may be simplified to $\hat{\mathbf{x}} = \mathbf{R}\mathbf{x}$. It is common practice to introduce a synthetic "truth" state, $\mathbf{x}$, which contains only one non-zero value, and then analyze the impulse

response, $\hat{\mathbf{x}}$, to describe the spatial resolution (9). For consistency with spatial resolution definitions in much of the GRACE literature, we define the spatial resolution as the best-fit Gaussian smoothing radius that best models the distance-decay of the synthetic impulse response.

Figure S1 provides examples of the impulse response behavior for monthly and stacked L1B regression mascons for high-signal regions (which have better resolution) at three different latitudes. This analysis results in best-fit Gaussian smoothing radii of 100-110 km for the stacked solutions and 235-350 km for the monthly, which corresponds to 5-10 factor improvements for the stacked estimates in terms of spatial area.

S.2 Statistical TWS trend attribution methods

S.2a Spatial lag model details

We ran continental-scale spatial autoregressive models in order to determine the relative influence of various anthropogenic and climatic variables on spatially variable TWS trends. Note that continental region boundaries were chosen based on Koppen-Geiger climate classification (11), rather than geopolitical boundaries.

Spatial lag models (SLMs), otherwise known as spatial autoregressive models, are multivariate regression techniques similar to ordinary least squares (OLS), but unlike basic linear regression, they explicitly account for spatial dependence by incorporating a weighted average of neighboring observations into the regression structure (12). A basic SLM model is given by

$$\boldsymbol{y} = \rho \boldsymbol{W} \boldsymbol{y} + \boldsymbol{X}\beta + \epsilon \qquad (8)$$

where $\mathbf{y}$ is the dependent variable, $\mathbf{W}$ is a spatial weights matrix defining neighborhood structure, and ρ is the autoregressive parameter, which typically ranges from -1 to 1. For our study, the dependent variable $\boldsymbol{y}$ is the collection of Level-1B regression TWS trends across global land mascons. Covariates $\boldsymbol{X}$ are anthropogenic and climatic variables aggregated to mascon spatial scale with area-weighted mean. Namely, $\boldsymbol{X}$ includes groundwater irrigated land area, surface water irrigated land area, 21st century reservoir impoundment, rainfed cropland area, deforested land area, glacial mass loss rate, precipitation anomaly during GRACE/FO, vapor pressure deficit anomaly during GRACE/FO, annual precipitation trend during GRACE/FO, and annual vapor pressure deficit trend during GRACE/FO.

In this study, we use a general extension in the SLM family called a spatial Durbin model (13), which jointly considers the spatial autocorrelation of the independent variables along with the dependent variable:

$$\boldsymbol{y} = \rho \boldsymbol{W} \boldsymbol{y} + \boldsymbol{X}\beta + \boldsymbol{W}\boldsymbol{X}\theta + \varepsilon \qquad (9)$$

In Equation 9, the θ coefficients represent the partial effect of neighboring $\boldsymbol{X}$ on local $\boldsymbol{y}$. This was important to include given that climate and land use variables are themselves spatially self-dependent, and because their impacts are not only felt locally but downstream as well. We found that restricting θ (for instance, the common factor restriction of spatial error models, another member of the SLM family, requires θ and β be the same sign) led to misspecification, since the local, direct impacts of a covariate may differ from the nonlocal feedbacks.

SLM results are sensitive to the spatial weights structure chosen. We constructed a spatial weights structure to best represent the spatial autocorrelation of the TWS trend map, as in previous econometric and geophysical studies (12, 14, 15). To do this, we fit correlograms of global land mascons using a distance-decay kernel with a Power-law function. Specifically, weights between locations *i* and *j* were defined as $w_{ij} = \frac{1}{1+\left(\frac{d}{h}\right)^{\alpha}}$ for $d_{ij} \leq 900$ km and set to zero

otherwise, where d_{ij} is the great-circle distance. Parameters h and α controlling the rate of decay were calibrated separately for each continental region, as shown in Figure S13, using Levenberg-Marquardt nonlinear least squares (16). A maximum autocorrelation distance of 900 km was chosen to reflect the point at which autocorrelation-decay of TWS trend land mascons begins to taper or approach a constant lower threshold (Figure 2c). We found that adjusting this value by $\pm$10% did not meaningfully change the outcomes of the continental spatial Durbin results (although standardized coefficients changed slightly in magnitude, their sign, significance, and relative contribution did not).

The choice of a globally uniform 900 km bandwidth reflects a deliberate decision to test a consistent spatial hypothesis across continents (namely, that TWS anomalies are spatially autocorrelated up to subcontinental scales) rather than allowing continent-specific bandwidths to conflate methodological variation with substantive geographic differences. However, spatial autoregressive model results could be sensitive to this bandwidth choice, as well as the method for $\boldsymbol{W}$ characterization, across continents with different spatial structures. To check this sensitivity, we replaced the $\boldsymbol{W}$ structure with another common autoregressive inference technique: defining spatial weights by the autocorrelation distance-decay of OLS residuals (15). We also set $w_{ij} = \frac{1}{1+\left(\frac{d}{h}\right)^{\alpha}}$ for $I < 0.05$ (representing minimal autocorrelation) and 0 otherwise, which allowed the maximum autocorrelation distance to vary by continent. Corresponding correlograms and β coefficients are reported in Figure S15; results did not differ greatly from those of the continental spatial Durbin models shown in Figure 3.

It should be noted that GRACE data are heavily autocorrelated in space, such that the ρ parameter frequently exceeded 0.8 or 0.9. The term $\rho\boldsymbol{W}\boldsymbol{y}$ represents spatial spillovers, allowing outcomes at one location to depend on outcomes nearby. Marginal effects are propagated through the spatial multiplier $\boldsymbol{S} = (\boldsymbol{I} - \rho\boldsymbol{W})^{-1}$, and direct impacts for a covariate *k* are derived from $\boldsymbol{S}\beta_k$. However, we chose not to report direct/indirect impacts, nor do we report lagged X coefficients θ. When the spatial autoregressive parameter ρ is this large, the empirical decomposition of total effects into direct and indirect components becomes numerically unstable and highly sensitive to the weight matrix specification (13), rendering the resulting estimates unreliable. Reporting the unlagged β coefficients is therefore the more defensible choice, as they capture the association between local predictor values and local TWS trends net of the spatially structured mean field absorbed by the autoregressive term, without invoking the unstable matrix-inverse operations that dominate effect decomposition at ρ approaching unity. Essentially, the β coefficients reported in Figure 3 represent how much the local TWS trend at a given location is associated with local variation in each predictor, after accounting for the tendency of nearby locations to have similar TWS trends. Non-local impacts were not calculated.

Spatial Durbin and spatial lag models include both original and spatially lagged versions of each predictor, which are by construction correlated with one another, making these models structurally more susceptible to multicollinearity than standard OLS regression. Multicollinearity occurs when two or more predictors are linearly correlated, inflating the variance of coefficient estimates and potentially rendering individual coefficients unstable or difficult to interpret even when overall model fit is good. Variance inflation factor (VIF) is a standard diagnostic that quantifies how much the variance of each coefficient is inflated relative to what it would be in the absence of collinearity (17). We computed VIF for all independent variable pairs across all continents, including for $\boldsymbol{W}\boldsymbol{y}$ (the spatially lagged dependent variable, representing the inverse-distance-weighted mean TWS trend of neighboring mascons, which enters the SDM as an additional predictor). VIF values typically ranged between 1 and 2, with a small number of predictor pairs exceeding 3 or 4; no value exceeded 5 (Table S7). These results are conventionally regarded as acceptable, reflecting only modest inflation that does not materially compromise the reliability or interpretability of individual parameter estimates. VIF values generally are of concern beyond ~5-10, at which point introduction of interaction terms may be appropriate (17).

S2b Lee's L index and local indicators of spatial association

Pearson's *r* quantifies the bivariate linear association between two variables observed at the same locations (18). However, this statistic neglects potential autocorrelation present in either variable, which can lead to significance inflation, and it also fails to consider how the lag in one variable may be correlated with the other (for instance, perturbations in one location may be correlated with phenomena downstream). Consider Pearson's *r*:

$$r_{X,Y} = \frac{\sum_i (x_i - \bar{x})(y_i - \bar{y})}{\sqrt{\sum_i (x_i - \bar{x})^2} \cdot \sqrt{\sum_i (y_i - \bar{y})^2}} \qquad (10)$$

We may define the autocorrelation of one variable, or the degree to which a value at one location is correlated with its neighbors, with:

$$I = \frac{n}{S_0} \cdot \frac{\sum_{i=1}^{n} \sum_{j=1}^{n} w_{i,j} (x_i - \bar{x})(x_j - \bar{x})}{\sum_{i=1}^{n} (x_i - \bar{x})^2} \qquad (11)$$

Lee's *L* index (18) is a bivariate measure of spatial association that integrates the logic of Pearson's correlation coefficient *r* (Equation 10) with the spatial autocorrelation framework of Moran's *I* (19) (Equation 11):

$$L_{X,Y} = \sqrt{\frac{\sum_i (\tilde{x}_i - \bar{x})^2}{\sum_i (x_i - \bar{x})^2}} \cdot \sqrt{\frac{\sum_i (\tilde{y}_i - \bar{y})^2}{\sum_i (y_i - \bar{y})^2}} \qquad (12)$$

In Equations 10-12, x_i and y_i are the observed values of the two variables at location i, and $\bar{x}$ and $\bar{y}$ are global mean values across all *n* locations. The spatially lagged values $\tilde{x}_i, \tilde{y}_i$ are the weighted averages of neighboring values: $\tilde{x}_i = \sum_j w_{ij} x_j$, $\tilde{y}_i = \sum_j w_{ij} y_j$, where w_{ij} are the spatial weights. The term S_0 in Equation 11 is the aggregate of all spatial weights.

To apply Lee's L to local indicators of spatial association, we compute the contribution of each individual location i to the overall spatial association, rather than averaging over all locations. Thus, local *L* is given by

$$L_{X,Y,i} = \sqrt{\frac{(\tilde{x}_i - \bar{x})^2}{\sum_k (x_k - \bar{x})^2}} \cdot \sqrt{\frac{(\tilde{y}_i - \bar{y})^2}{\sum_k (y_k - \bar{y})^2}} \qquad (13)$$

Essentially, each factor measures the ratio of the variance of the spatially lagged variable to the variance of the original variable. Their product gives a global measure of spatial association analogous to a bivariate Moran's *I*, scaled similarly to Pearson's correlation coefficient.

Figures

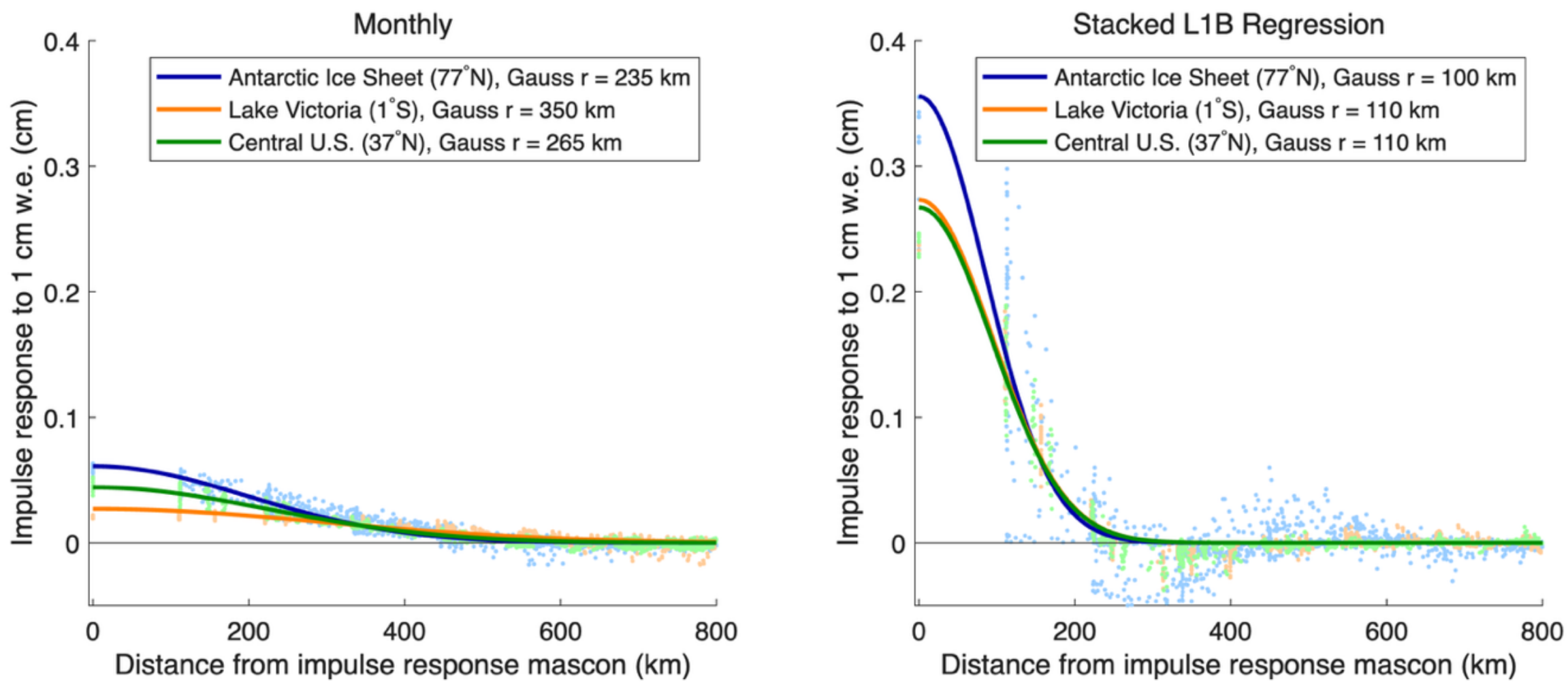

Figure S1: Impulse response as a function of distance from artificial signal introduced of 1 cm water equivalent. Each point represents mascons at a given distance bin, shown for pulses introduced at a mascon in the Antarctic Ice Sheet, in Lake Victoria, and in the central U.S. Results are shown for TWS trends derived from GSFC monthly mascons (left) and the Level-1B stacked regression (right).

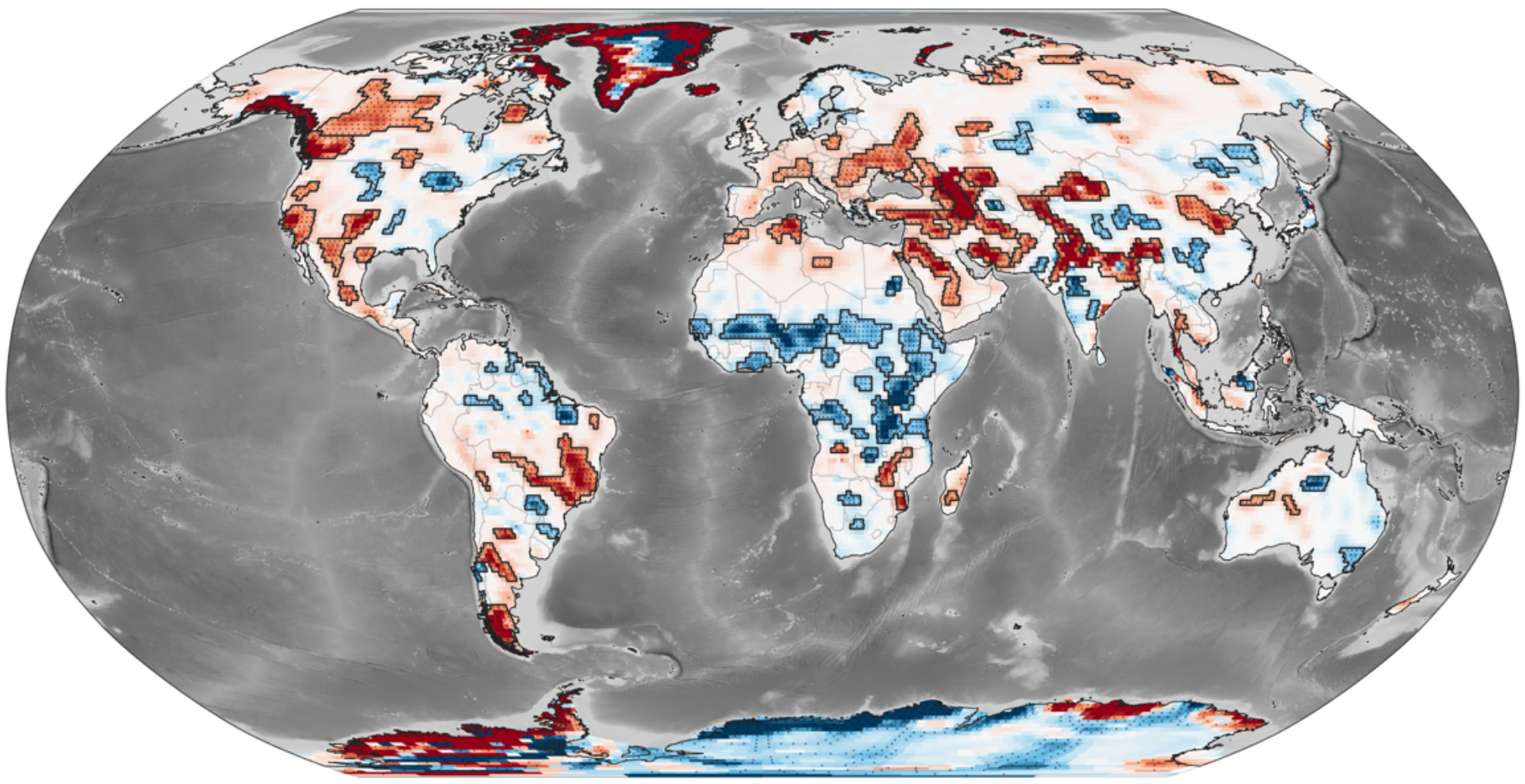

Fig. S2. L1B regression noise uncertainty. Map of L1B Stacked TWS anomaly trends from 2002 to 2025 across GSFC 1-arc degree equal-area land mascons. Stippling indicates mascons where trend magnitudes exceed $\boldsymbol{\sigma_{land}} = \frac{\mathbf{STDEV(GSFC-GOCO)}}{\sqrt{\mathbf{2}}} = \mathbf{0.70}$ cm yr^{-1} L1B Stacked solution noise, indicating a significant trend. The mascon groups are regions where trend magnitudes exceed noise and GIA uncertainty and where human activity impacts the TWS trends.

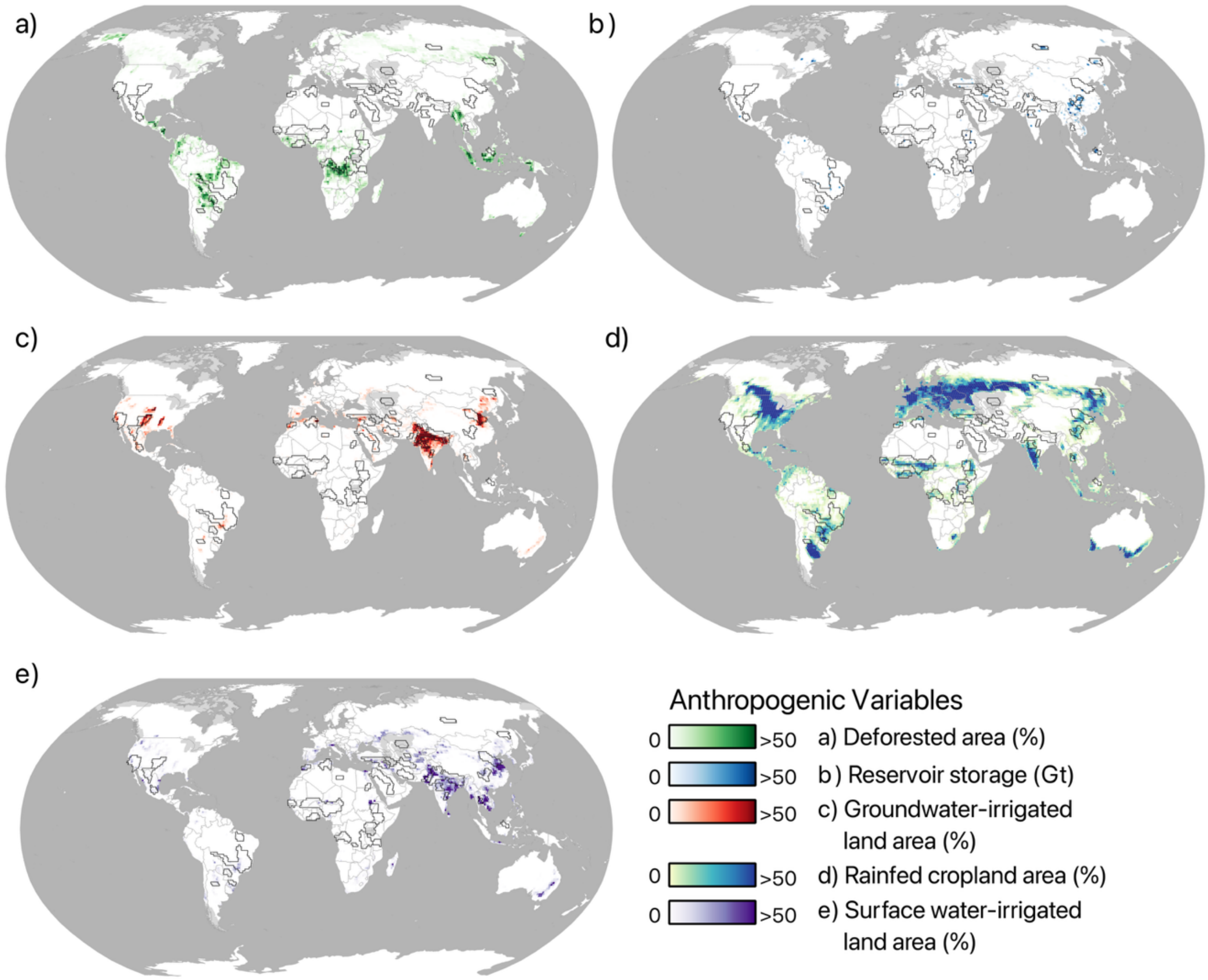

Fig. S3: Anthropogenic spatial lag model covariates. (**A**) Forested land area reductions from 1960 to 2019, from the HIstoric Land Dynamics Assessment + (HILDA +) expressed as percentage of land area (20). (**B**) Newly impounded (21st century) reservoir storage (21). All variables were aggregated to 1-arc-degree mascon area using area-weighted average. (**C**) Percent of land area equipped for groundwater irrigation, circa 2005, created from the Food and Agriculture Organization (FAO) Global Map of Irrigated Areas (GMIA) (22) and HILDA+ datasets. (**D**) Global rainfed cropland (23) expressed as percentage of land area. (**E**) Percent of land area equipped for surface water irrigation, circa 2005, created from GMIA and HILDA+ datasets.

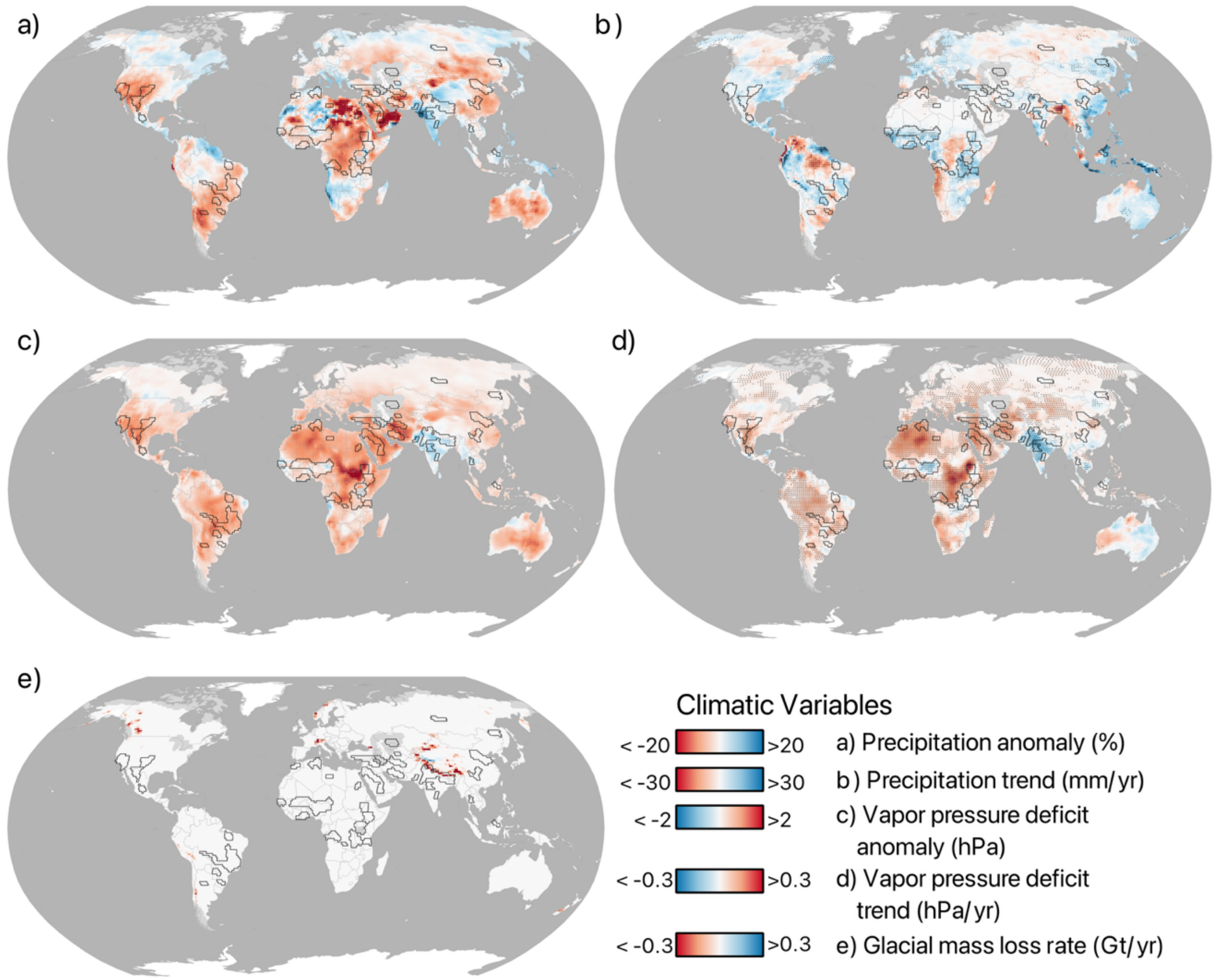

Fig. S4 Climatic spatial lag model covariates. (**A**) Precipitation during the GRACE and GRACE-FO observation period (2002 – 2025) shown as percent of long term normal mean annual precipitation (1979-2025) (24). (**B**) Trend in annual precipitation during the GRACE and GRACE-FO observation period (mm yr^{-1}). (**C**) Vapor pressure deficit anomaly during the GRACE and GRACE-FO observation period relative to long-term mean (hPa). (**D**) Trend in annual mean vapor pressure deficit during the GRACE/FO observation period (hPa yr^{-1}). Trend significance ($p<0.05$, Mann-Kendall trend test) is indicated by stippling. (**E**) Mean annual glacial mass loss rate (25) All variables were aggregated to 1-arc-degree mascon area using area-weighted average.

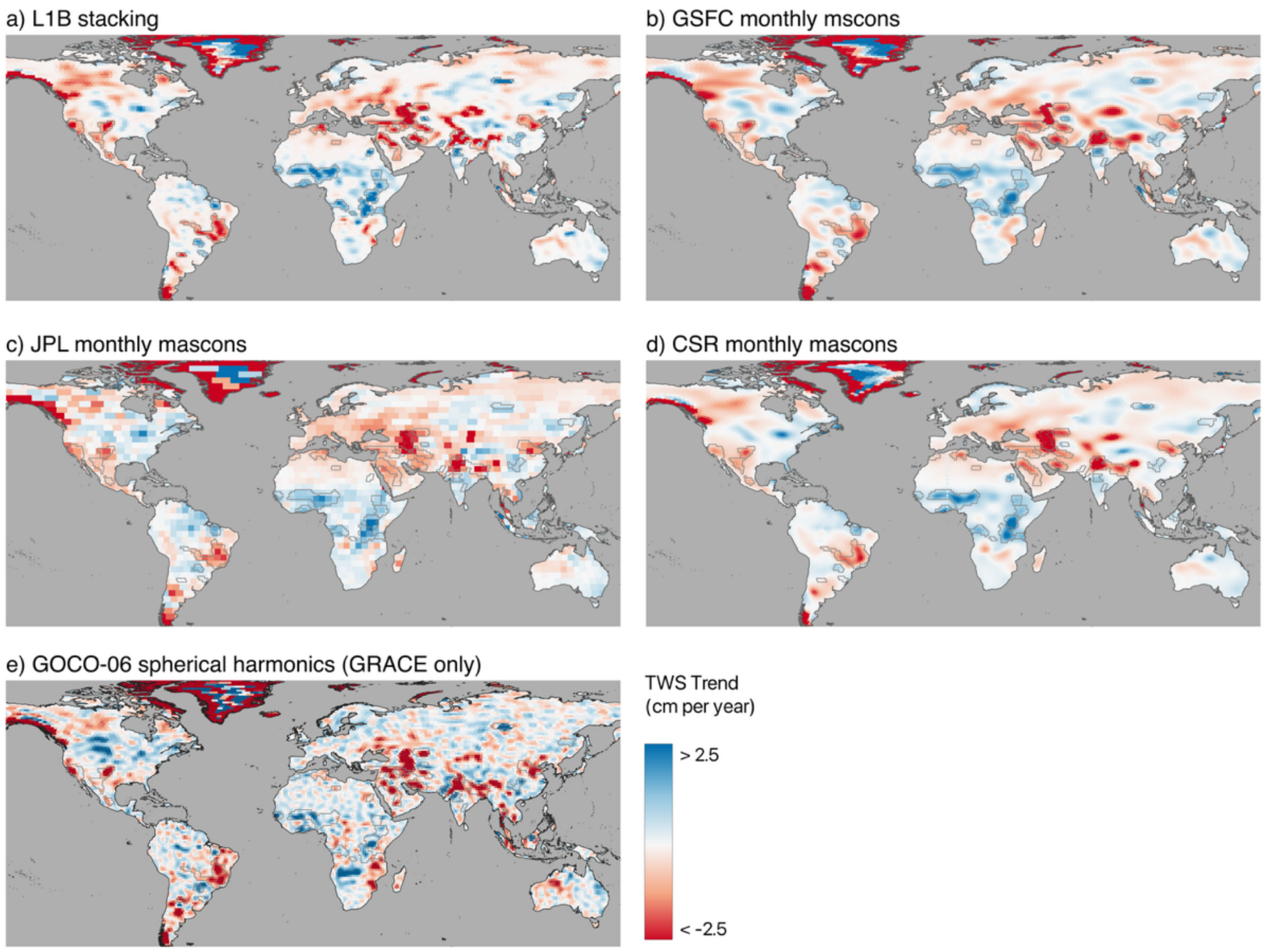

Fig. S5 Global GRACE-FO TWS trend comparisons between mascon products. 2002 – 2025 TWS trends evaluated across global land area using regression of **A**) Level-1B intersatellite range data, **B**) GSFC monthly mascons, **C**) JPL monthly mascons, and **D**) CSR monthly mascons. We also compare to the 2002 – 2016 terrestrial mass change rate from the GOCO-06 spherical harmonic product evaluated at GSFC mascon locations (**E**).

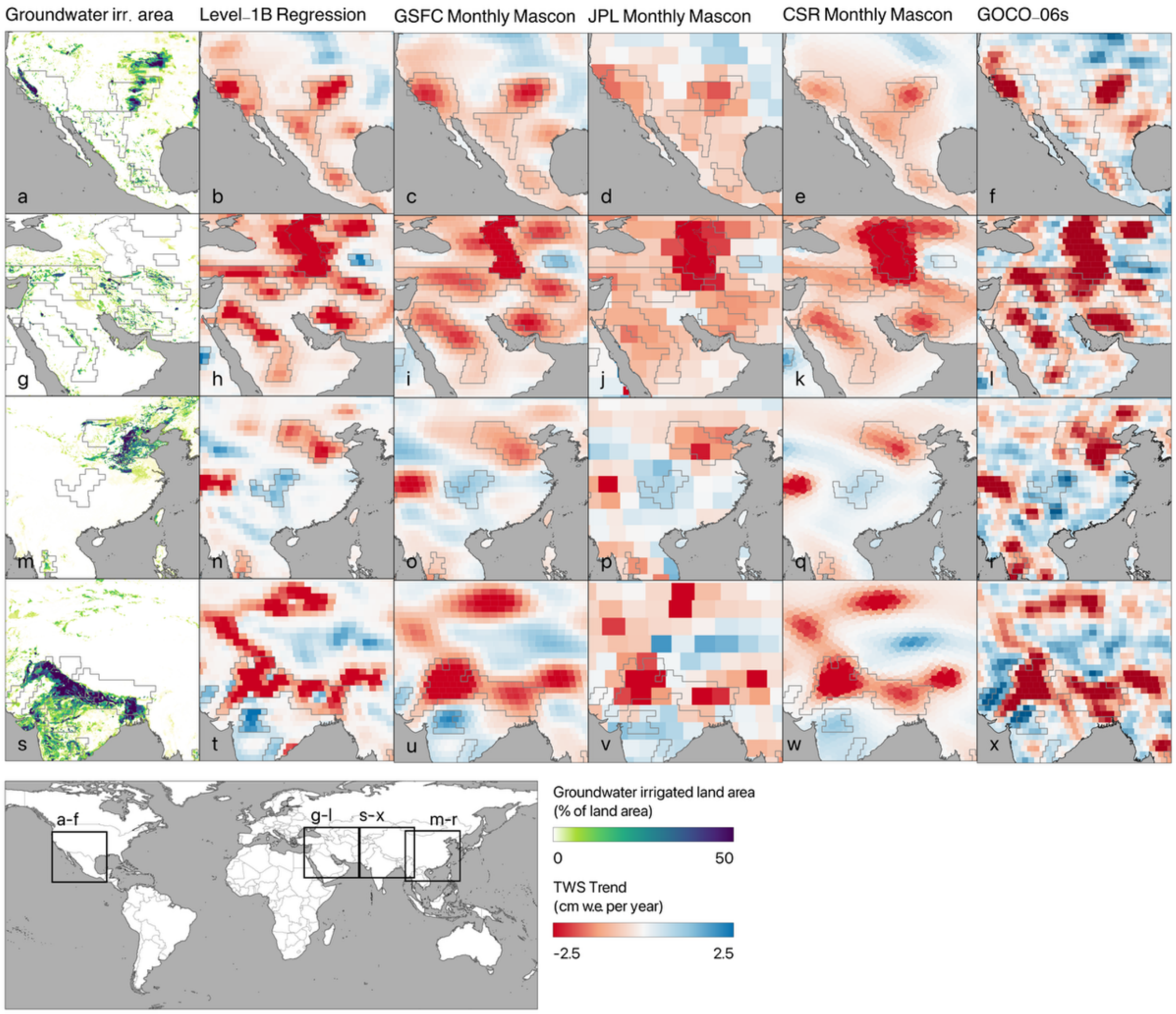

Fig. S6: TWS trend comparisons between GRACE products for heavily irrigated regions. Percent of irrigated land equipped for groundwater irrigation (22) (**A**, **G**, **M**, **S**), and TWS trends found from different GRACE/FO TWS anomaly products, over North America (**A-F**), the Middle East (**G-L**), the China (**M-R**), and Northern India (**S-X**). TWS trends from the GRACE and GRACE-FO periods (2002 – 2025) are shown from the L1B stacking approach (**B**, **H**, **N**, **T**) and from linear regression of monthly TWS anomalies from GSFC (9) (**C**, **I**, **O**, **U**), JPL (26) (**D**, **J**, **P**, **V**), and CSR (27) (**E**, **K**, **Q**, **W**) mascons. The GOCO-06s (28) spherical harmonic TWS trend was evaluated at GSFC mascons for the GRACE period 2002 – 2016.

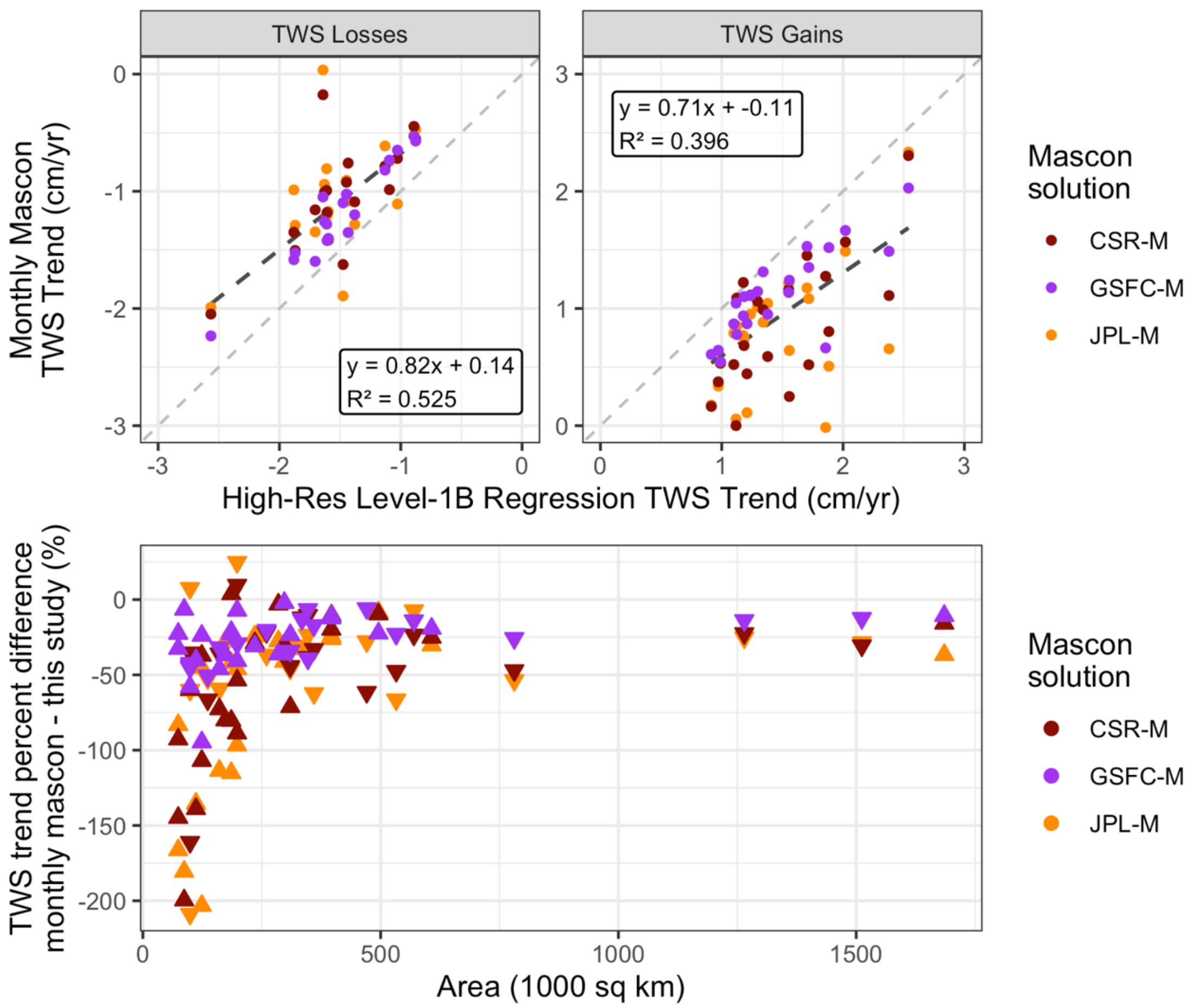

Fig. S7: TWS trend comparisons between GRACE products. Comparisons between region-averaged Level-1B regression of TWS trends and region-averaged TWS trends from linear regression of monthly TWS anomalies from JPL mascons (26), CSR mascons (27), and GSFC mascons (9). In the lower panel, upward (downward) arrows indicate the high-resolution L1B-Stacked solution estimated a positive (negative) TWS trend (storage gains and losses, respectively), and percent difference for each region is shown as a function of region area (x-axis).

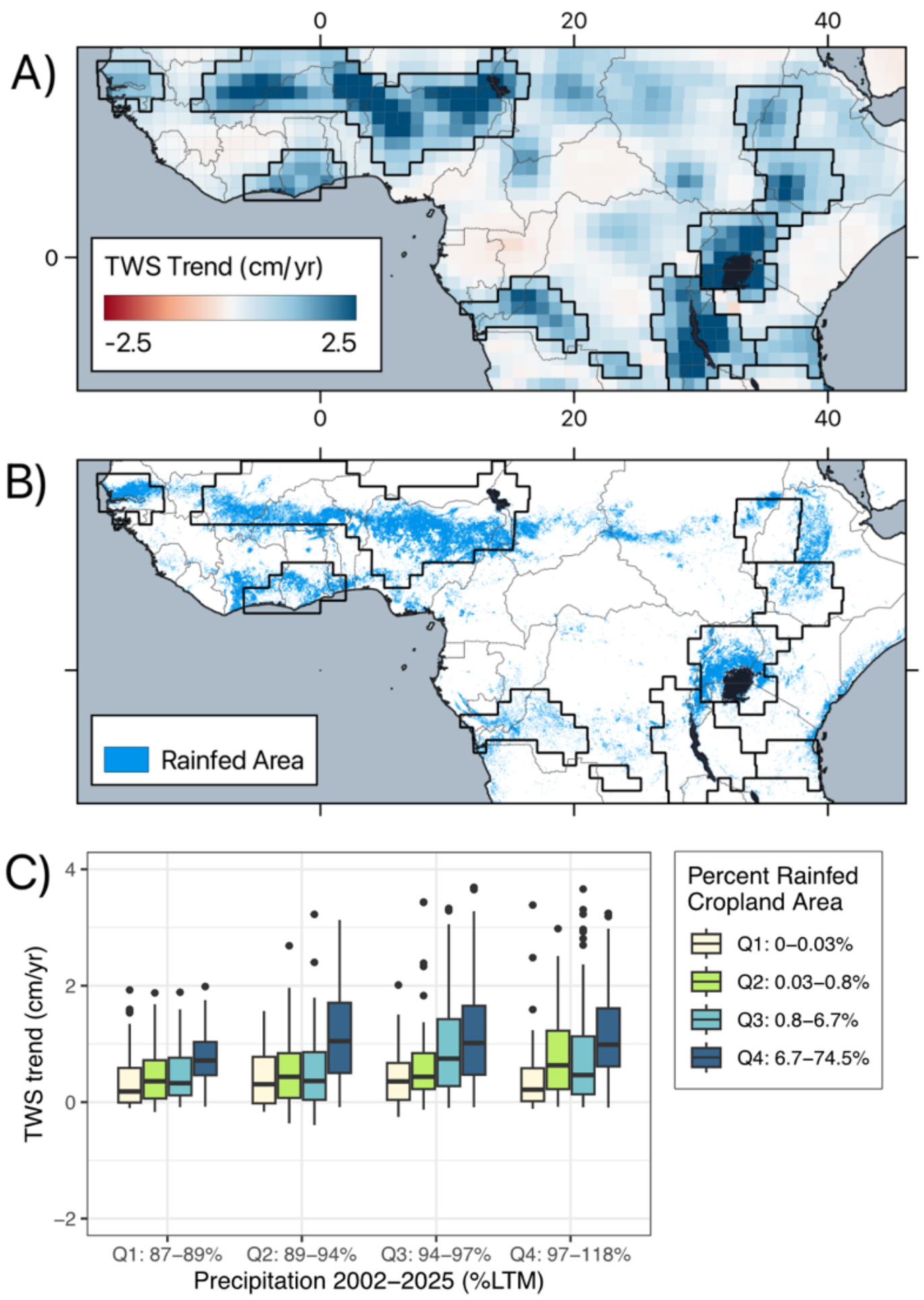

Fig. S8: TWS trends and rainfed cropland over Sub-Saharan Africa. A) TWS trends from 2002 – 2025 from the Level-1B regression method. **B**) Map of rainfed cropland area (23). (**C**) Boxplots of TWS trends for individual mascons within the land area shown in (**A**) and (**B**), grouped by quantiles of mascon-aggregated precipitation during the observation period (percent of 40 year normal) (x-axis) (24) and percent of rainfed cropland mascon area (color groupings).

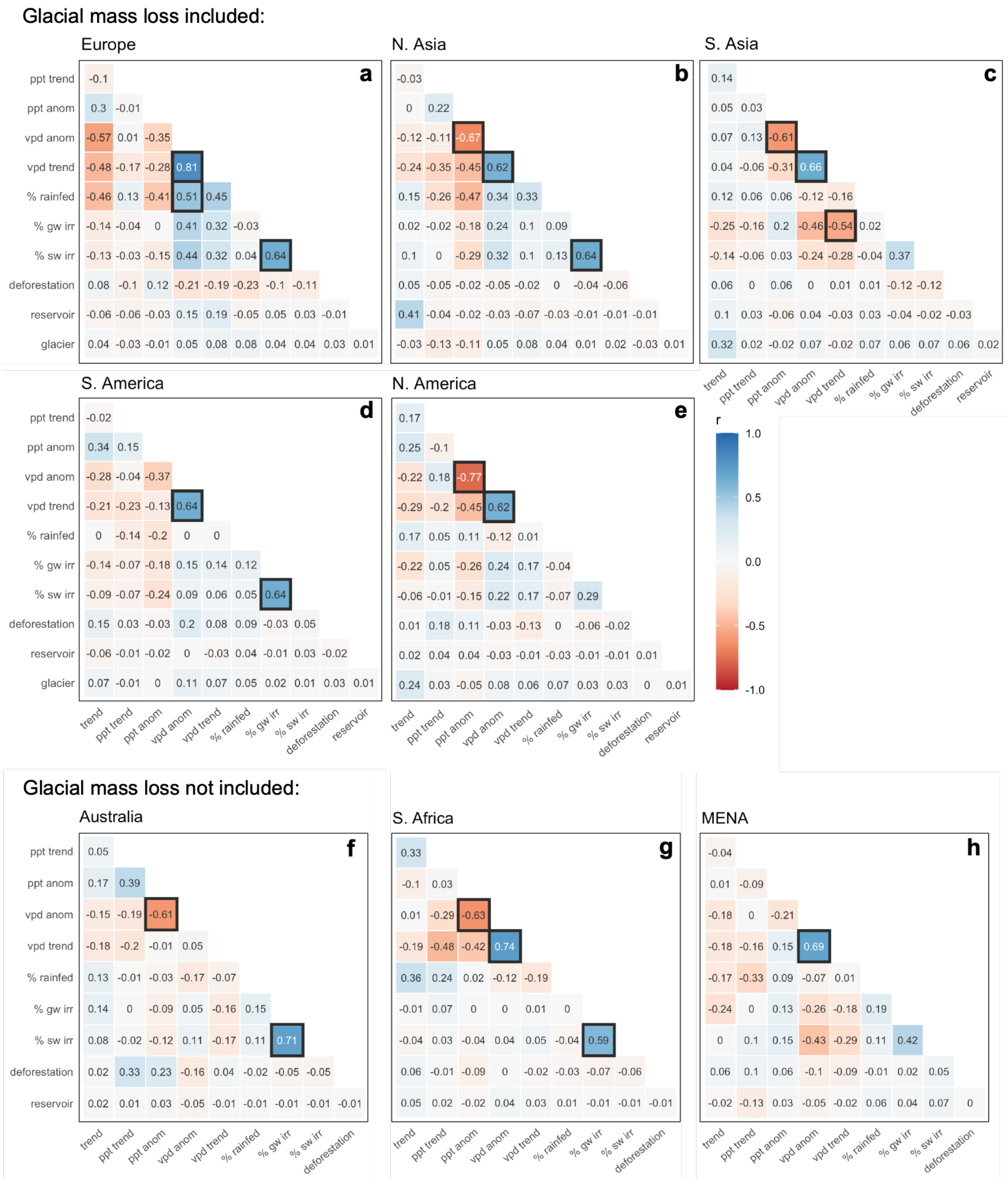

Figure S9: Pearson's r correlations between dependent (trend) and independent variables in spatial autoregressive models, for continental regions shown in Figure S14. Bolded cells indicate cross-correlations exceeding a magnitude of r=0.5. Covariates (top to bottom / left to right) include trends and anomalies in annual precipitation and vapor pressure deficit, percent rainfed irrigated land area, groundwater irrigated land area, and surface water irrigated land area, deforested land area, total impounded reservoir storage, and glacial melt rate. Dependent variable ("trend") is TWS trend during 2002-2025.

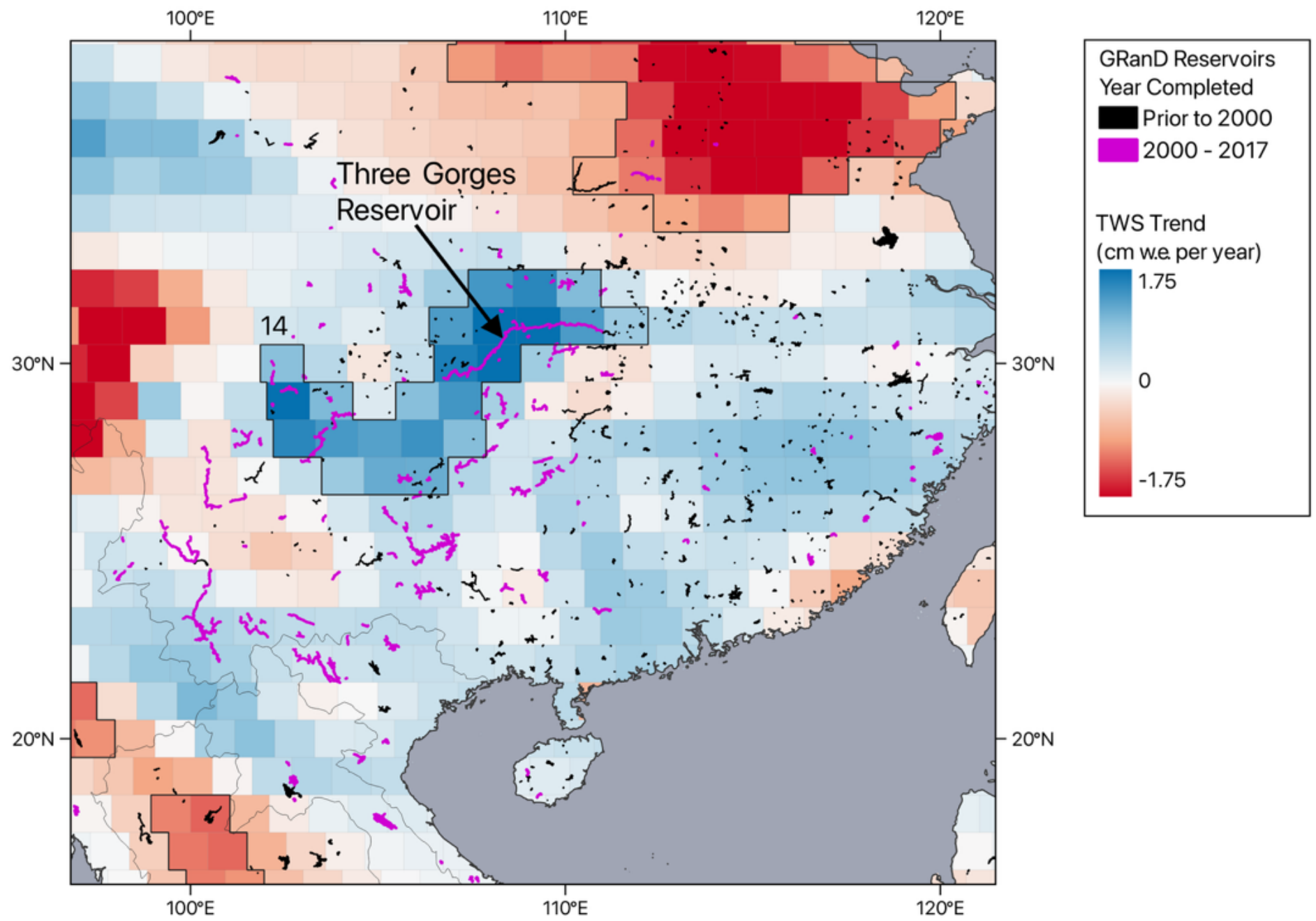

Fig. S10: TWS trends (2002 – 2025) and reservoirs in southeast China. TWS trends found using the L1B stacking approach for monthly TWS anomalies from the GRACE/FO period (2002 – 2025). Reservoirs (21) built prior to the GRACE/FO period are shown in black and reservoirs built and filled after 2000 are shown in pink. The Three Gorges Reservoir overlays the three mascons with largest positive TWS trends within Region 14.

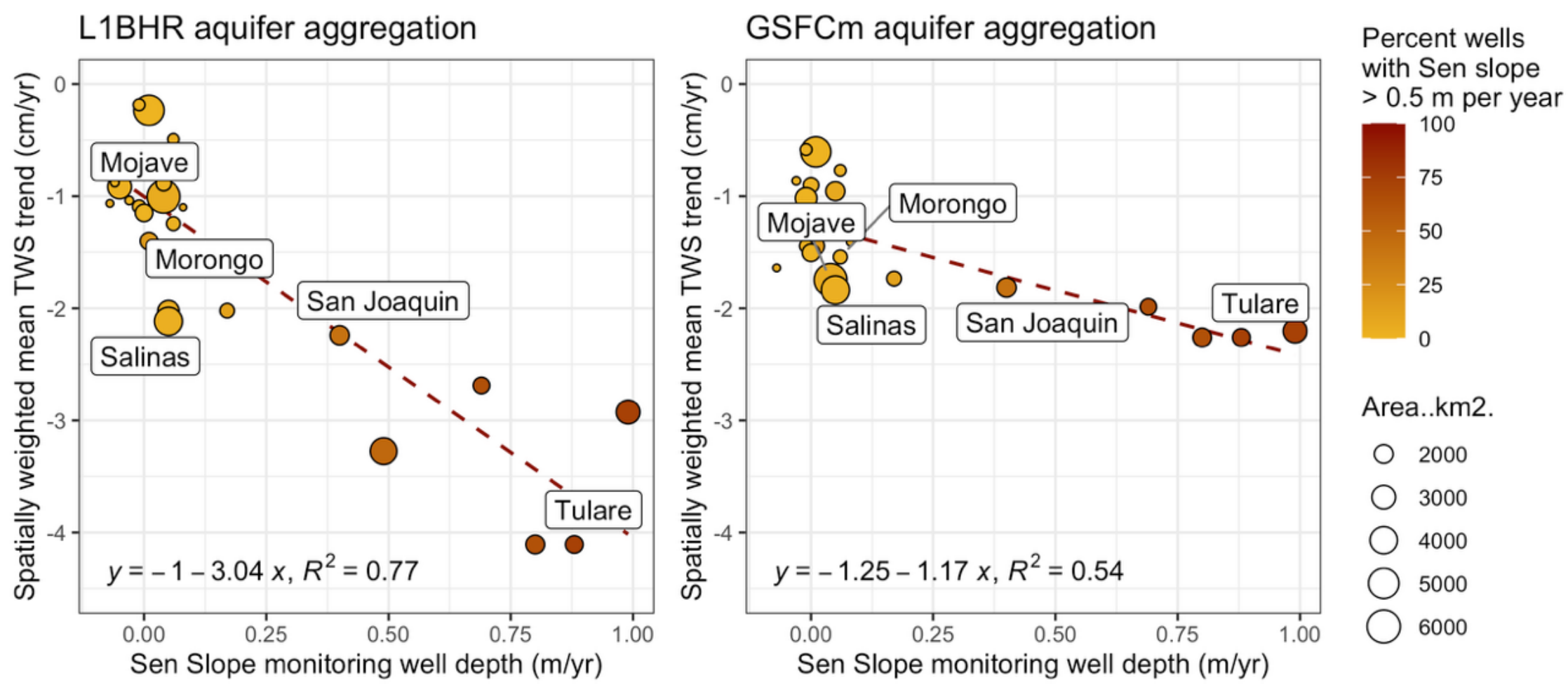

Figure S11: Comparison between aquifer-aggregate median Sen slope of monitoring well depth from Jasechko and Perrone (2021). Left: comparisons with TWS trends from Level-1B regression (this study). Right: comparisons with TWS trends from regression of GSFC monthly mascons. Aquifers selected were only those greater than 1000 km^2 in area and containing an observation well density of at least 1 well per every 10 km^2. No attempt was made to select wells with similar observation timeframe as the GRACE and GRACE-FO periods.

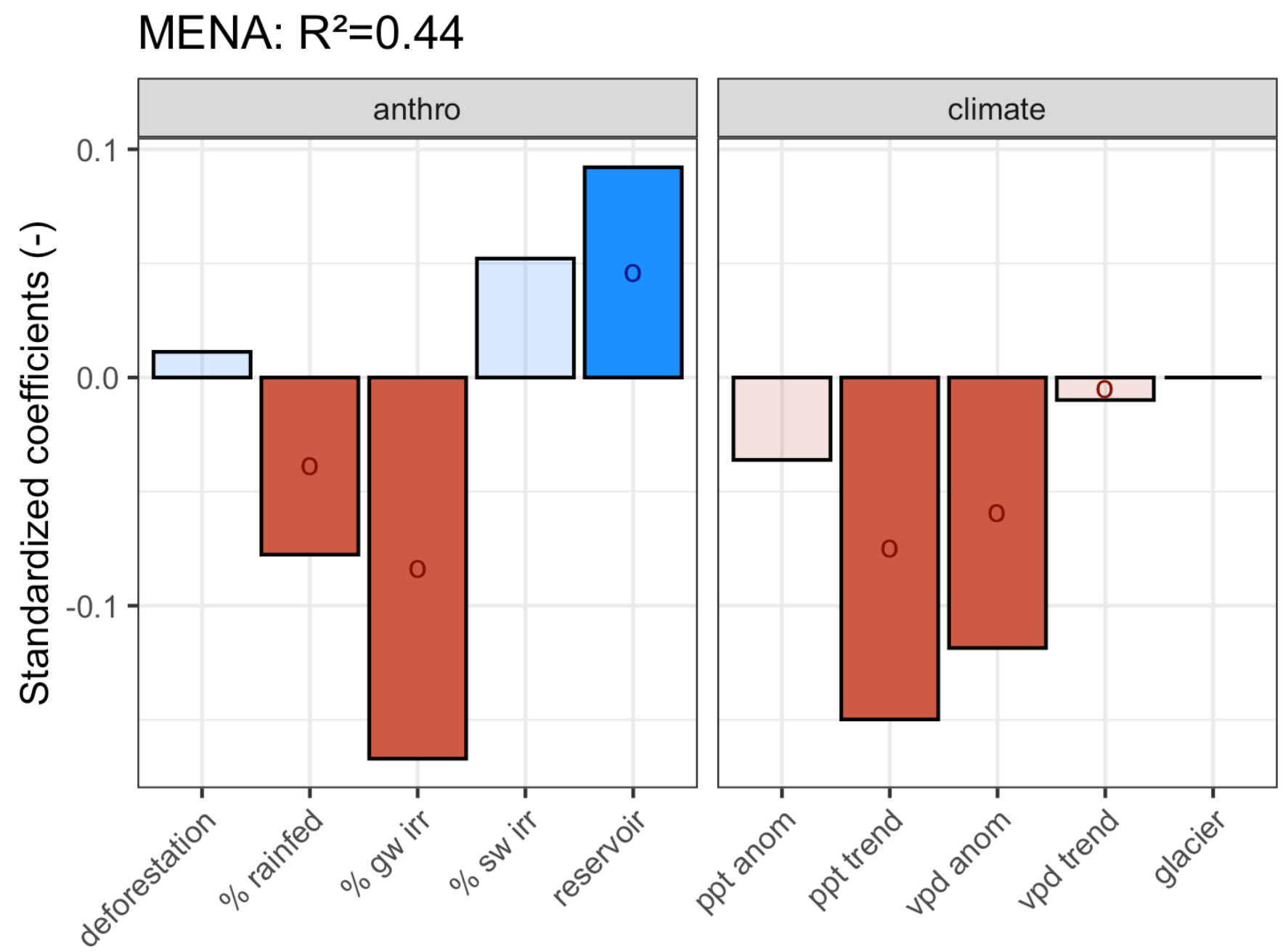

Figure 12: Standardized coefficients from spatial Durbin model for MENA region when Toshka Lake volume is added to reservoir impoundment covariate.

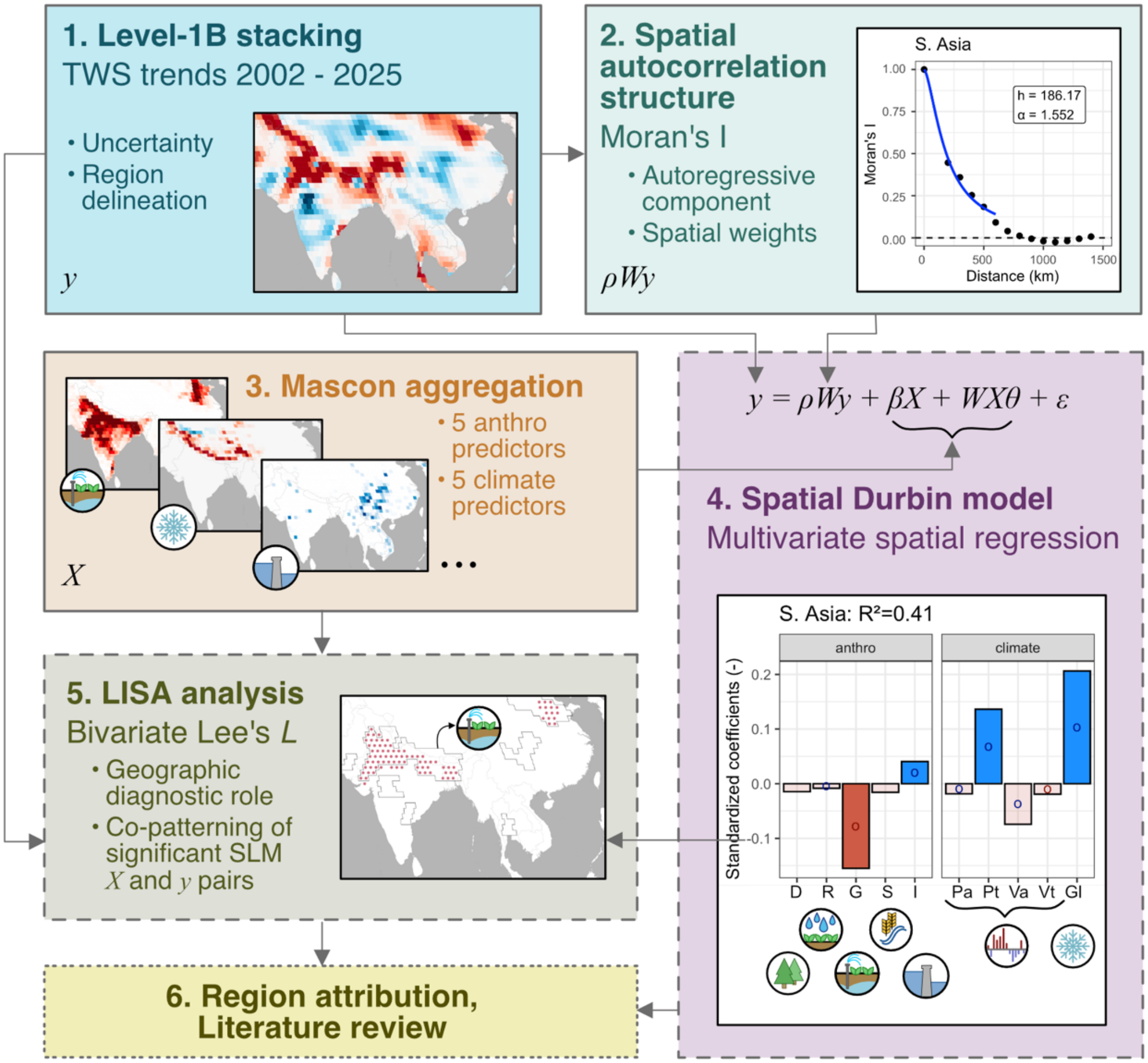

Figure S13: Schematic of the methodological workflow used in this study.

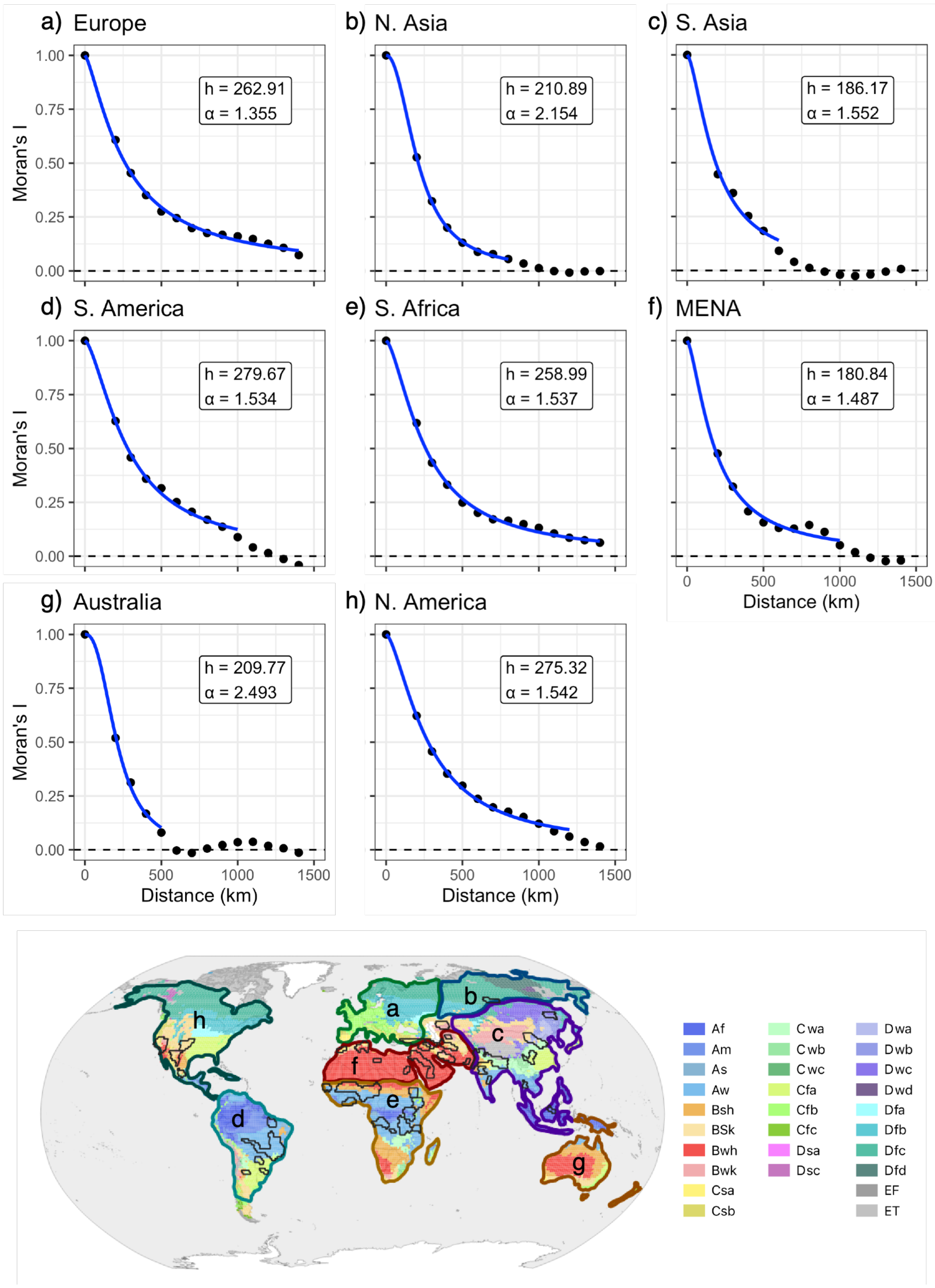

Figure S14: Correlograms (Moran's I for progressive distance bins) of TWS trend maps for 8 continental scale regions (boundaries chosen roughly using Koppen-Geiger classification (11)). The blue lines indicate best power-law fit, with corresponding parameters displayed for each continent, used to construct the spatial weights matrix in the spatial Durbin models.

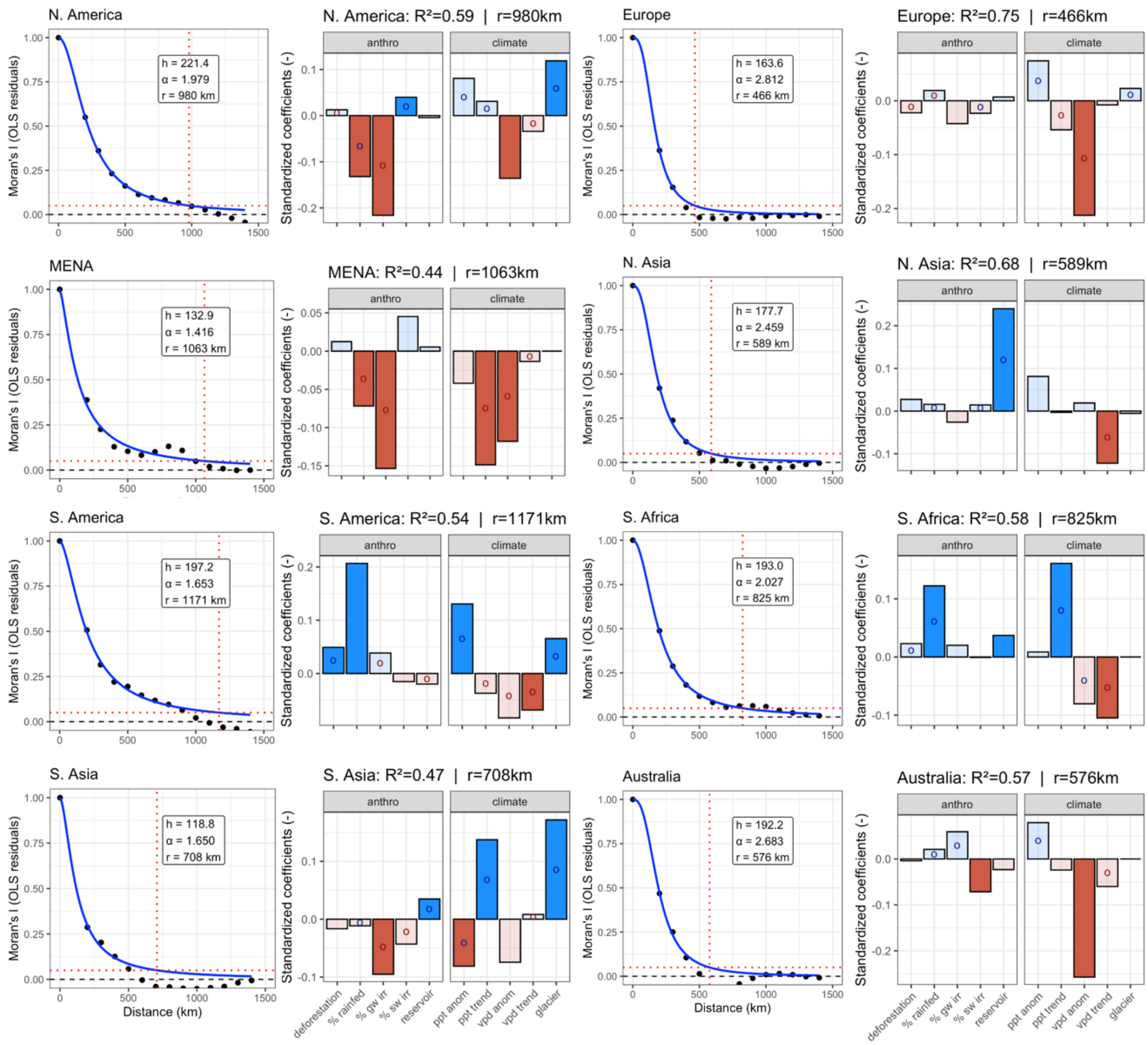

Figure S15: Testing SDM sensitivity to spatial weights matrix (W) construction. Spatial weights were characterized by best-fit power-law function up to the corresponding distance at which I=0.05. Pairs of OLS residual correlograms (left) and resulting SDM standardized coefficients (right) are shown for each continental region (corresponding to regions shown in Figure S13). SDM coefficients may be interpreted as in Figure 3.

Tables

Table S1. Summary of GRACE products used in this study

Product	Years used	Satellite source observations	Level regression performed	Application in this study
L1B-Stacked	2002 – 2025	GRACE/FO	Level-1B	Reported global TWS trends
L1B-Stacked	2002 - 2016	GRACE	Level-1B	Uncertainty
GOCO-06s	2002 - 2016	GRACE, SLR, GNSS kinematic orbits, GOCE gravity gradiometer	Level-1B	Uncertainty
JPL-Mascon	2002 - 2025	GRACE/FO	Level-3	Comparison
CSR-Mascon	2002 - 2025	GRACE/FO	Level-3	Comparison
GSFC-Mascon	2002 - 2025	GRACE/FO	Level-3	Comparison

Table S2: L1B Stacked TWS trends, noise and GIA uncertainty for regions of interest. Area-weighted mean of TWS trends (2002 – 2022) over regions of interest, found using the L1B stacking approach, along with region area. The total uncertainty is defined as twice the root sum square of noise uncertainty and GIA error.

Region	Location	HR L1B-Stacked TWS Trend (this study) (cm/yr)	Area (1000 sq km)	Total TWS Trend Uncertainty (cm/yr)	Noise Uncertainty (cm/yr)	GIA Error (cm/yr)	Uncertainty to trend magnitude ratio	HR L1B-Stacked TWS Trend (Gt/yr)	Total TWS Trend Uncertainty (Gt/yr)	Total TWS Change 2002-2025 (Gt)	Total TWS Change Uncertainty (Gt)
1	West Sudanian Savanna	1.7	1685	0.13	0.06	0.02	0.08	28.7	2.18	672.1	51
2	Senegal and the Gambia	1.18	198	0.35	0.17	0.01	0.30	2.35	0.69	55	16.2
3	Southern Ghana	1.34	297	0.28	0.14	0.01	0.21	3.99	0.85	93.4	19.8
4	Lake Victoria	2.54	495	0.23	0.11	0.03	0.09	12.58	1.12	294.6	26.3
5	Central Parana Basin	1.38	173	0.4	0.19	0.08	0.29	2.39	0.7	56	16.4
6	Brazil South Atlantic Region	0.91	112	0.51	0.23	0.11	0.56	1.02	0.57	23.9	13.4
7	Maranhao Brazil	1.55	235	0.32	0.16	0.01	0.21	3.65	0.75	85.5	17.6
8	Bateke Plateau DRC	1.3	396	0.25	0.12	0.03	0.19	5.13	1	120.2	23.4
9	Lunda Plateau DRC	1.21	74	0.57	0.28	0.04	0.47	0.9	0.43	21	10
10	Lake Tanganyika Region	2.02	607	0.21	0.1	0.04	0.10	12.26	1.29	287.2	30.3
11	Great Ethiopian Renaissance Dam	1.12	285	0.29	0.14	0	0.26	3.2	0.83	75	19.4
12	Boguchany Reservoir	2.38	161	0.59	0.19	0.22	0.25	3.83	0.94	89.6	22.1
13	Malaysian Reservoirs	1.72	124	0.44	0.22	0.01	0.26	2.13	0.54	49.8	12.8
14	Central Yangtze	1.1	310	0.36	0.14	0.12	0.33	3.41	1.13	79.8	26.4

15	Eastern Tanzania	1.18	186	0.37	0.18	0.04	0.31	2.19	0.68	51.3	16
16	Omo and Rift Valley Basins	1.24	397	0.25	0.12	0.01	0.20	4.91	0.98	115	23
17	Southeastern Russia	0.97	198	0.49	0.17	0.17	0.51	1.92	0.97	45	22.8
18	Lower Indus River	1.12	87	0.58	0.26	0.12	0.52	0.97	0.5	22.7	11.8
19	Karakum Desert	1.56	74	0.62	0.28	0.13	0.40	1.16	0.46	27.1	10.9
20	Madhya Pradesh India	1.88	186	0.4	0.18	0.09	0.21	3.5	0.75	81.9	17.5
21	Southeastern India	0.99	99	0.51	0.25	0.07	0.52	0.98	0.51	23	11.9
22	Central Thailand	-1.03	99	0.51	0.25	0.06	0.50	-1.02	0.5	-23.8	11.8
23	North China Plain	-1.38	570	0.36	0.1	0.15	0.26	-7.86	2.04	-184.1	47.8
24	Northern India	-2.56	1265	0.28	0.07	0.12	0.11	-32.44	3.59	-759.7	84
25	Northern NWSAS	-1.45	310	0.29	0.14	0.04	0.20	-4.48	0.89	-105	20.9
26	Morocco	-0.89	136	0.42	0.21	0.02	0.47	-1.22	0.57	-28.5	13.4
27	Central Saudi Arabia	-1.63	781	0.21	0.09	0.06	0.13	-12.72	1.65	-298	38.7
28	Fertile Crescent	-1.61	533	0.27	0.11	0.09	0.17	-8.58	1.45	-201	34.1
29	Western Iran	-1.48	198	0.4	0.17	0.1	0.27	-2.92	0.79	-68.5	18.5
30	Southern Iran	-1.88	360	0.33	0.13	0.1	0.18	-6.77	1.18	-158.5	27.7
31	Northern Iran	-1.6	334	0.35	0.13	0.12	0.22	-5.34	1.19	-125.1	27.8
32	Southwestern US	-1.43	471	0.27	0.11	0.08	0.19	-6.75	1.29	-158.1	30.2
33	Southern High Plains	-1.71	347	0.38	0.13	0.14	0.22	-5.92	1.31	-138.5	30.8
34	Chihuahua Mexico	-1.09	347	0.33	0.13	0.1	0.30	-3.79	1.13	-88.9	26.5
35	Mexico City	-1.13	161	0.4	0.19	0.05	0.35	-1.82	0.64	-42.6	15

36	Brazilian Highlands	-1.61	1512	0.16	0.06	0.05	0.10	-24.3	2.48	-569.1	58
37	GMMR Libya	-0.88	99	0.49	0.25	0.03	0.56	-0.87	0.49	-20.4	11.5
38	Aral Sea	-1.87	260	0.41	0.15	0.14	0.22	-4.87	1.07	-114	25
39	Mar Chiquita Lake	-1.64	99	0.53	0.25	0.1	0.32	-1.63	0.53	-38.1	12.4
40	Nasser and Toshka Lakes	1.86	124	0.45	0.22	0.04	0.24	2.3	0.55	53.9	12.9

Table S3: Irrigation and land use over regions of interest. Area-weighted mean of percent rainfed land area (c. 2005) from the Global Rainfed, Irrigated and Paddy Cropland (GRIPC) dataset (23); percent of land experiencing shift from 1960 forest to 2019 non-forest from the Historic Land Dynamics Assessment + (HILDA +) (20); number of dams, and dam impoundment in Gt summed from all reservoirs filled after 2000 within the region, according to the GRANDE dataset (21); HILDA+ percent cropland area in 2019 (20); percent land equipped for groundwater and surface water irrigation in 2005 (22).

Region	Location	HR L1B-Stacked TWS Trend (this study) (cm/yr)	GRIPC % Rainfed Crop Land Area (c. 2005)	Change in HILDA forested extent (% land area since 1960)	21st Century Reservoir Impoundments, GRANDE (Gt)	Number of 21st Century Reservoirs Built, GRANDE	HILDA Cropland Extent 2019 (% land area)	HILDA/FAO % of land equipped for groundwater irrigation (c. 2000)	HILDA/FAO % of land equipped for surface water irrigation (c. 2000)
1	West Sudanian Savanna	1.7	17.2	-0.4	0.4	2	37	0.2	1.1
2	Senegal and the Gambia	1.18	15.4	1.2			19.6	0	0.2
3	Southern Ghana	1.34	18.1	-4.5	12.6	1	13.4	0	0.2
4	Lake Victoria	2.54	15.4	-5.6			34.8	0	0
5	Central Parana Basin	1.38	25.1	-17.5			45.2	0.4	0.9
6	Brazil South Atlantic Region	0.91	31.7	-4.1	0.3	1	48.6	0.4	2.4
7	Maranhao Brazil	1.55	6.7	-14.9			3	0.6	0.4
8	Bateke Plateau DRC	1.3	9.2	-23.7	0.6	1	2.2	0	0.1
9	Lunda Plateau DRC	1.21	0.6	-54.1			1.4	0	0
10	Lake Tanganyika Region	2.02	3.1	-13.7			10.9	0	0.1
11	Great Ethiopian Renaissance Dam	1.12	8.2	-3.8	74	1	34.4	0	0.8
12	Boguchany Reservoir	2.38	0.2	-0.8	58.2	1	0	0	0

13	Malaysian Reservoirs	1.72	1.6	-6.5	55.8	2	4.2	0	0
14	Central Yangtze	1.1	13.9	7.7	77.3	29	14	0	4.8
15	Eastern Tanzania	1.18	3.7	-1.4	34	1	14.3	0.1	0.4
16	Omo and Rift Valley Basins	1.24	7.5	-2.9	15.5	2	14	0	0.2
17	Southeastern Russia	0.97	18.5	-9.8	20.9	1	6.3	0.2	0.1
18	Lower Indus River	1.12	0	0			48.8	4.7	44.1
19	Karakum Desert	1.56	0	0			0	0	0
20	Madhya Pradesh India	1.88	11.5	4.9	2.4	3	80.3	48.5	18.1
21	Southeastern India	0.99	10	13.2			57.5	22.4	20.5
22	Central Thailand	-1.03	12.2	4.7	0.2	1	69	6.7	42.5
23	North China Plain	-1.38	10.1	2.3	1.3	4	43.6	18.6	13.7
24	Northern India	-2.56	0.6	2.8	3.9	2	53.6	29.2	23.6
25	Northern NWSAS	-1.45	5.6	0.8	1.3	4	18.5	2.4	1
26	Morocco	-0.89	12.4	1.4	1.1	2	51.1	13.4	14.7
27	Central Saudi Arabia	-1.63	0	0			3.5	3.2	0.3
28	Fertile Crescent	-1.61	7.4	1.9	8.8	11	32.2	5.5	9.6
29	Western Iran	-1.48	3.2	2.2	2.6	10	29.7	9.6	9.5
30	Southern Iran	-1.88	0	4.9	3.1	7	4.1	3.3	0.8
31	Northern Iran	-1.6	0.3	1.1	0.3	2	8.3	4.7	2.8
32	Southwestern US	-1.43	0.1	1.5	1	1	8.7	5.3	3.2
33	Southern High Plains	-1.71	2.4	1.8			29.1	20.4	3.1
34	Chihuahua Mexico	-1.09	0.2	-0.1			6.2	3.6	1.7
35	Mexico City	-1.13	6.1	0			25.8	6.9	7
36	Brazilian Highlands	-1.61	7.3	-1.8	25.3	15	20.1	1.4	2.9

37	GMMR Libya	-0.88	0	0	0.9	0.9	0
38	Aral Sea	-1.87	0	0.4	0.2	0	0.2
39	Mar Chiquita Lake	-1.64	7.7	-3.2	14.5	0.8	2.4
40	Nasser and Toshka Lakes	1.86	0	0	0.5	0.1	0.5

Table S4: Precipitation normals and trends over regions of interest. Area-weighted mean annual precipitation from 1982 – 2025 from ERA5 (24) reanalysis 0.25-degree grids. Precipitation during the GRACE/FO period (2002 – 2025) and the decade prior (1992 – 2002) are shown as percent deviation from long term (1982 – 2025) normal precipitation. Trends in precipitation during 1982 – 2025 and 2002 – 2025 periods are the Sen slope of annual precipitation, with bolded values indicating significance from Mann Kendall trend test (*P*<0.05).

Region	Location	HR L1B-Stacked TWS Trend (cm/yr)	1979 - 2025 Mean Annual Precipitation (mm)	2002 - 2025 Mean Annual Precipitation (mm)	2002-2025 Annual Precipitation Anomaly (mm)	2002 - 2025 Anomaly (% LTM)	Prior decade, 1992 - 2002 Anomaly (% LTM)	Annual Precipitation Trends 1979 - 2025 (mm/yr)	Annual Precipitation Trends 2002 - 2025 (mm/yr)
1	West Sudanian Savanna	1.7	572	561	-11	-2	3	-0.13	**4.76**
2	Senegal and the Gambia	1.18	674	661	-13	-2	0	-0.07	**7.23**
3	Southern Ghana	1.34	1335	1308	-27	-2	-6	-2.14	**10.1**
4	Lake Victoria	2.54	1579	1516	-63	-4	-3	**-6.36**	8.1
5	Central Parana Basin	1.38	1634	1520	-114	-7	9	**-6.94**	8.11
6	Brazil South Atlantic Region	0.91	1838	1728	-110	-6	10	**-7.57**	-8.68
7	Maranhao Brazil	1.55	1649	1616	-33	-2	1	-1.91	0.32
8	Bateke Plateau DRC	1.3	1490	1401	-89	-6	7	**-6.37**	0.14
9	Lunda Plateau DRC	1.21	1480	1391	-89	-6	3	**-7.61**	3.43
10	Lake Tanganyika Region	2.02	1614	1549	-65	-4	-3	**-4.29**	**11.23**
11	Great Ethiopian Renaissance Dam	1.12	1345	1264	-81	-6	5	**-6.09**	4.66
12	Boguchany Reservoir	2.38	513	513	0	0	-2	-0.05	-1.84
13	Malaysian Reservoirs	1.72	3349	3449	100	3	-2	**7.34**	12.08
14	Central Yangtze	1.1	1669	1586	-83	-5	5	**-5.43**	-0.14
15	Eastern Tanzania	1.18	943	915	-28	-3	-4	-1.91	**13.34**

16	Omo and Rift Valley Basins	1.24	1112	1090	-22	-2	-4	-2.28	4.69
17	Southeastern Russia	0.97	799	783	-16	-2	-3	-0.79	**5.77**
18	Lower Indus River	1.12	168	178	10	6	3	0.24	0.51
19	Karakum Desert	1.56	128	131	3	2	-4	-0.03	-0.26
20	Madhya Pradesh India	1.88	972	1030	58	6	-3	**3.98**	1.24
21	Southeastern India	0.99	1049	1112	63	6	-7	**4.85**	0.16
22	Central Thailand	-1.03	1432	1446	14	1	2	1.5	-0.12
23	North China Plain	-1.38	602	584	-18	-3	4	-1.46	-0.81
24	Northern India	-2.56	1278	1291	13	1	2	1.23	-3.63
25	Northern NWSAS	-1.45	254	259	5	2	-9	-0.5	**-3.33**
26	Morocco	-0.89	431	427	-4	-1	-3	-1.12	-4.44
27	Central Saudi Arabia	-1.63	58	54	-4	-7	15	-0.29	-0.43
28	Fertile Crescent	-1.61	461	452	-9	-2	0	-0.83	2.77
29	Western Iran	-1.48	495	500	5	1	-1	0.08	-0.35
30	Southern Iran	-1.88	187	178	-9	-5	11	-0.43	1.11
31	Northern Iran	-1.6	270	259	-11	-4	2	-0.91	1.66
32	Southwestern US	-1.43	337	310	-27	-8	11	-1.47	0.95
33	Southern High Plains	-1.71	544	490	-54	-10	3	**-4.54**	1.15
34	Chihuahua Mexico	-1.09	378	348	-30	-8	-13	**-2.53**	1.02
35	Mexico City	-1.13	921	930	9	1	-2	0.77	0.36
36	Brazilian Highlands	-1.61	1340	1273	-67	-5	2	**-6.35**	0.33
37	GMMR Libya	-0.88	15	14	-1	-8	2	-0.13	-0.23
38	Aral Sea	-1.87	188	179	-9	-5	2	**-1.04**	-1.07
39	Mar Chiquita Lake	-1.64	927	816	-111	-12	13	**-8.43**	-3.72
40	Nasser and Toshka Lakes	1.86	2	2	0	-16	27	0	0.06

Table S5: Vapor pressure deficit normals and trends over regions of interest. Same as table S4, but for ERA5 vapor pressure deficit.

Region	Location	HR L1B-Stacked TWS Trend (cm/yr)	1979 - 2025 Mean Annual VPD (hPa)	2002 - 2025 Mean Annual VPD (hPa)	2002-2025 Annual VPD Anomaly (hPa)	2002 - 2025 Anomaly (% LTM)	Prior decade, 1992 - 2002 Anomaly (% LTM)	Annual VPD Trends 1979 - 2025 (hPa/yr)	Annual VPD Trends 2002 - 2025 (hPa/yr)
1	West Sudanian Savanna	1.7	22.5	22.7	0.2	1.1	-1.9	0.02	-0.02
2	Senegal and the Gambia	1.18	17.2	17.1	-0.1	-0.7	0.8	-0.01	0.03
3	Southern Ghana	1.34	7.4	7.7	0.3	3.6	-2.2	**0.02**	**0.03**
4	Lake Victoria	2.54	9.2	9.7	0.5	5.8	-2.3	**0.05**	0.04
5	Central Parana Basin	1.38	8.1	8.8	0.7	8.5	-8.7	**0.05**	0.02
6	Brazil South Atlantic Region	0.91	5.6	5.8	0.2	3.3	-6.8	**0.01**	0.02
7	Maranhao Brazil	1.55	8.6	9.2	0.6	7.2	-6.1	**0.05**	0.03
8	Bateke Plateau DRC	1.3	6.3	6.9	0.6	10.2	-9.9	**0.05**	**0.07**
9	Lunda Plateau DRC	1.21	9.4	10.0	0.6	6.4	-2.5	**0.05**	**0.02**
10	Lake Tanganyika Region	2.02	8.6	9.0	0.4	4.8	1.6	**0.03**	0
11	Great Ethiopian Renaissance Dam	1.12	17	18.2	1.2	6.9	-5.8	**0.11**	**0.16**
12	Boguchany Reservoir	2.38	2.7	2.8	0.1	2.3	4	**0.01**	0
13	Malaysian Reservoirs	1.72	4.3	4.4	0.1	3.4	1.7	**0.01**	0.01
14	Central Yangtze	1.1	3.9	4.1	0.2	6.2	-4.7	**0.02**	0
15	Eastern Tanzania	1.18	8.9	9.3	0.4	4.6	-0.3	**0.03**	0
16	Omo and Rift Valley Basins	1.24	12.6	13.2	0.6	4.9	-0.8	**0.06**	0.04
17	Southeastern Russia	0.97	2.6	2.7	0.1	4.3	-1.6	**0.01**	0
18	Lower Indus River	1.12	20.1	19.5	-0.6	-3.2	1.8	**-0.06**	**-0.07**
19	Karakum Desert	1.56	15	15.6	0.6	4.1	-5.3	**0.05**	**0.08**

20	Madhya Pradesh India	1.88	16.4	15.8	-0.6	-3.4	0.1	**-0.07**	**-0.14**
21	Southeastern India	0.99	14.2	13.9	-0.3	-2	-0.5	**-0.04**	-0.04
22	Central Thailand	-1.03	10.6	10.4	-0.2	-2.3	2.7	-0.02	0
23	North China Plain	-1.38	6.6	7.0	0.4	5.4	-1.5	**0.03**	**0.03**
24	Northern India	-2.56	9.8	9.6	-0.2	-2.2	-0.7	**-0.03**	**-0.05**
25	Northern NWSAS	-1.45	14.3	15.0	0.7	4.6	-1.7	**0.07**	**0.1**
26	Morocco	-0.89	8.4	9.1	0.7	8.1	-7.4	**0.06**	**0.1**
27	Central Saudi Arabia	-1.63	24.8	25.8	1.0	3.9	-2.2	**0.09**	**0.1**
28	Fertile Crescent	-1.61	14.6	15.3	0.7	5.1	-2.8	**0.07**	**0.08**
29	Western Iran	-1.48	9.4	10.1	0.7	7.4	-1.5	**0.07**	**0.07**
30	Southern Iran	-1.88	20.2	21.1	0.9	4.5	-4.2	**0.08**	**0.09**
31	Northern Iran	-1.6	14.6	15.7	1.1	7.6	-4.3	**0.1**	**0.1**
32	Southwestern US	-1.43	13.3	14.1	0.8	5.8	-5.1	**0.06**	**0.06**
33	Southern High Plains	-1.71	9.2	9.8	0.6	6.6	-4.8	**0.05**	0.04
34	Chihuahua Mexico	-1.09	13.5	14.6	1.1	7.8	0	**0.09**	**0.08**
35	Mexico City	-1.13	8.3	8.6	0.3	3.4	-0.4	**0.03**	**0.06**
36	Brazilian Highlands	-1.61	9.4	10.2	0.8	8.3	-5.6	**0.06**	**0.06**
37	GMMR Libya	-0.88	21.2	21.9	0.7	3.1	-1.4	**0.06**	**0.07**
38	Aral Sea	-1.87	9.1	9.9	0.8	8.7	-9.2	**0.06**	**0.07**
39	Mar Chiquita Lake	-1.64	10.2	11.1	0.9	8.8	-9.3	**0.07**	**0.09**
40	Nasser and Toshka Lakes	1.86	27.7	28.7	1.0	3.7	-2.2	**0.08**	**0.05**

Table S6: Comparisons between TWS trends found from various GRACE products. TWS trends during the GRACE and GRACE-FO observation period (2002 – 2025) found from the L1B Stacking approach and from linear regression of monthly TWS anomalies from GSFC (9), CSR (27), and JPL (26) mascons. The average of the three monthly mascon products (GSFC, CSR, and JPL) is also shown. Trends were area-weighted over regions of interest and bolded values indicate that the difference between the L1B Stacked product and the mean of other GRACE solution exceeds L1B Stacked TWS trend uncertainty. Also shown are the L1B Stacked TWS trend and GOCO-06s (28) spherical harmonic TWS trend from 2002 – 2016. The uncertainty of the L1B Stacked product from 2002 – 2016 is assumed to be roughly equivalent to uncertainty from 2002 – 2025.

Region	Area (1000 sq km)	HR L1B-Stacked TWS Trend (cm/yr)	HR L1B-Stacked TWS Trend Uncertainty (cm/yr)	GSFC Monthly Mascon TWS Trend (cm/yr)	CSR Monthly Mascon TWS Trend (cm/yr)	JPL Monthly Mascon TWS Trend (cm/yr)	Mean Monthly Mascon TWS Trend (cm/yr)	Monthly Mascon TWS Trend Uncertainty (cm/yr)	Mean Monthly Mascon TWS Trend (Gt/yr)	HR L1B-Stacked TWS Trend (Gt/yr)	Mean Monthly Mascon TWS Trend Magnitude Bias (%)	HR L1B-Stacked TWS Trend 2002 - 2016 (cm/yr)	GOCO-06s TWS Trend 2002 - 2016 (cm/yr)
1	1685	1.7	0.13	1.53	1.45	1.18	**1.39**	0.18	**23.37**	28.7	-19	-0.04	0.27
2	198	1.18	0.35	1.1	0.68	0.74	0.84	0.23	1.66	2.35	-29	0.32	0.42
3	297	1.34	0.28	1.31	0.99	0.88	1.06	0.22	3.15	3.99	-21	-0.03	-0.04
4	495	2.54	0.23	2.03	2.3	2.33	**2.22**	0.17	**11**	12.58	-13	-0.04	0.16
5	173	1.38	0.4	0.95	0.59	1.04	**0.86**	0.24	**1.49**	2.39	-38	-1.11	-0.81
6	112	0.91	0.51	0.61	0.16	0.18	**0.32**	0.25	**0.35**	1.02	-65	0.48	0.62
7	235	1.55	0.32	1.14	1.16	1.22	**1.17**	0.04	**2.76**	3.65	-24	0.04	0.24
8	396	1.3	0.25	1.14	1.06	1.01	1.07	0.07	4.24	5.13	-17	-0.77	-0.97
9	74	1.21	0.57	0.87	0.44	0.11	**0.47**	0.38	**0.35**	0.9	-61	-1.4	-0.73
10	607	2.02	0.21	1.67	1.57	1.49	**1.58**	0.09	**9.57**	12.26	-22	-1.02	-0.74
11	285	1.12	0.29	0.78	1.09	0.85	0.91	0.16	2.58	3.2	-19	-0.25	0.26
12	161	2.38	0.59	1.49	1.11	0.66	**1.09**	0.42	**1.75**	3.83	-54	-0.01	0.34
13	124	1.72	0.44	1.35	0.52	1.08	**0.98**	0.42	**1.22**	2.13	-43	-5.89	-3.34
14	310	1.1	0.36	0.87	0.52	0.79	**0.73**	0.18	**2.25**	3.41	-34	0.09	0.13
15	186	1.18	0.37	0.94	1.22	0.77	0.98	0.23	1.82	2.19	-17	-0.01	0.21
16	397	1.24	0.25	1.11	1.11	0.95	1.06	0.09	4.19	4.91	-15	-0.22	0.03
17	198	0.97	0.49	0.64	0.37	0.34	**0.45**	0.17	**0.89**	1.92	-54	0.83	0.49

18	87	1.12	0.58	1.05	0	0.06	**0.37**	0.59	**0.32**	0.97	-67	-0.67	-0.47
19	74	1.56	0.62	1.24	0.25	0.64	**0.71**	0.50	**0.53**	1.16	-54	-0.6	-0.02
20	186	1.88	0.4	1.52	0.8	0.51	**0.94**	0.52	**1.75**	3.5	-50	-0.59	-0.56
21	99	0.99	0.51	0.55	0.53	0.53	0.54	0.01	0.53	0.98	-46	-0.11	-0.27
22	99	-1.03	0.51	-0.65	-0.72	-1.11	-0.83	0.25	-0.82	-1.02	-20	-0.67	-0.08
23	570	-1.38	0.36	-1.2	-1.09	-1.28	-1.19	0.10	-6.78	-7.86	-14	-0.42	-0.49
24	1265	-2.56	0.28	-2.23	-2.05	-1.99	**-2.09**	0.12	**-26.43**	-32.44	-19	-0.03	0
25	310	-1.45	0.29	-1.02	-0.92	-0.91	**-0.95**	0.06	**-2.94**	-4.48	-34	-0.31	-0.11
26	136	-0.89	0.42	-0.53	-0.45	-0.52	-0.5	0.04	-0.68	-1.22	-44	0.02	0.1
27	781	-1.63	0.21	-1.26	-1.01	-0.94	**-1.07**	0.17	**-8.36**	-12.72	-34	-0.08	0.02
28	533	-1.61	0.27	-1.28	-0.99	-0.81	**-1.03**	0.24	**-5.47**	-8.58	-36	-1.11	-0.78
29	198	-1.48	0.4	-1.1	-1.62	-1.89	-1.54	0.40	-3.05	-2.92	4	0.47	0.47
30	360	-1.88	0.33	-1.58	-1.35	-0.99	**-1.31**	0.30	**-4.7**	-6.77	-31	-0.13	0.06
31	334	-1.6	0.35	-1.4	-1.42	-1.17	-1.33	0.14	-4.45	-5.34	-17	-0.31	0.03
32	471	-1.43	0.27	-1.35	-0.76	-1.08	**-1.06**	0.30	**-5.01**	-6.75	-26	-0.26	-0.54
33	347	-1.71	0.38	-1.6	-1.16	-1.35	-1.37	0.22	-4.75	-5.92	-20	0.77	0.6
34	347	-1.09	0.33	-0.73	-0.99	-0.74	-0.82	0.15	-2.85	-3.79	-25	-0.92	-0.72
35	161	-1.13	0.4	-0.82	-0.79	-0.61	-0.74	0.11	-1.19	-1.82	-35	-1.25	-1.21
36	1512	-1.61	0.16	-1.42	-1.18	-1.21	**-1.27**	0.13	**-19.2**	-24.3	-21	-0.1	0.24
37	99	-0.88	0.49	-0.57	-0.55	-0.47	-0.53	0.05	-0.53	-0.87	-40	0.93	0.82
38	260	-1.87	0.41	-1.53	-1.5	-1.29	**-1.44**	0.13	**-3.75**	-4.87	-23	-0.14	-0.13
39	99	-1.64	0.53	-1.05	-0.18	0.03	**-0.4**	0.57	**-0.4**	-1.63	-76	-0.19	0.57
40	124	1.86	0.45	0.66	1.27	-0.01	**0.64**	0.64	**0.79**	2.3	-66	-0.05	0.21

Table S7: Variance inflation factors for independent variables. Wy indicates the y variable (TWS trend) smoothed by the spatial weights structure (i.e., it is the autocorrelation term). Values approaching the lower limit at which multi-collinearity would be a model specification concern (usually >5) are bolded in red. However, these values are still not large enough to constitute an interaction term or other change to model specification.

Standardized independent variables	Europe	North Asia	South Asia	Sub-Saharan Africa	North America	MENA	South America	Australia
Precipitation anomaly	1.28	2.17	1.74	1.80	2.85	1.30	1.57	2.02
Precipitation trend	1.18	1.26	1.17	1.55	1.36	1.23	1.16	1.37
Vapor pressure deficit anomaly	**4.71**	2.62	2.93	**3.15**	**3.98**	2.70	2.22	1.73
Vapor pressure deficit trend	**3.57**	2.11	2.27	2.68	2.17	2.53	1.91	1.22
Rainfed cropland area	1.95	1.57	1.11	1.18	1.18	1.20	1.11	1.13
Groundwater irrigated land area	1.93	1.72	1.87	1.54	1.16	1.33	1.79	2.05
Surface water irrigated land area	1.86	1.89	1.23	1.54	1.13	1.45	1.78	2.06
Deforested land area	1.09	1.05	1.04	1.02	1.07	1.03	1.13	1.16
Reservoir impoundment mass	1.07	1.06	1.02	1.00	1.01	1.04	1.01	1.01
Glacial melt rate	1.02	1.03	1.17	-	1.07	-	1.02	-
Wy	2.87	1.42	1.55	1.48	1.55	1.20	1.42	1.29

Table S8: Literature comparisons of TWS trends in groundwater irrigated areas. Summary of literature review for groundwater pumping regions. Studies for comparison are those published after 2020 with at least 12 years of GRACE data (more than half our study timeframe) used for trend estimation. To illustrate differences resulting from study area size, we applied the referenced (reported or calculated) trend in cm/yr to this study's land area size in the last column to achieve comparable Gt/yr. We also supply comparisons to Rodell et al., 2018 (29) in red (not included in average bias calculation).

Region	Region name	Reference	Reference location	GRACE Product Used	Timeframe	N. years	Area (1000 sq km)	Trend (Gt/yr)	Unc. (Gt/yr)	Trend (cm/yr)	Unc. (cm/yr)	Trend applied over our study area (Gt/yr)	Average rate bias by recent publications (%) (Rodell et al. 2018 not included)
**22**	**Central Thailand**	**This study**		**HR L1B-Stacked (This Study)**	**2002 - 2025**	**24**	**99**	**-1.02**	**0.5**	**-1.03**	**0.51**	**-1.02**	**-5.3%**
		(30)	Chao Phraya River Basin		2002 - 2020	19	160	-1.79	0.08	-1.12	0.05	**-1.11**	
		(31)	Global, Chao Phraya River Basin	ITSG-GRACE2016, JPL-M	2003 - 2015	13	158	**-1.31**	**0.49**	-0.83	0.31	**-0.82**	
**23**	**North China Plain**	**This study**		**HR L1B-Stacked (This Study)**	**2002 - 2025**	**24**	**570**	**-7.86**	**2.04**	**-1.38**	**0.36**	**-7.87**	**-11.6%**
		(32)	North China Plain	JPL-M, CSR-M, JPL-SH, CSR-SH, GFZ-SH	2004 - 2020	17	542	**-5.64**	**0.27**	-1.04	0.05	**-5.93**	
		(33)	North China Plain	JPL-M, CSR-M, GSFC-M	2002 - 2020	19	370	**-5.18**	**0.52**	-1.40	0.14	**-7.98**	

		(29)	North China Plain	JPL-M	2002 - 2016	15	876	-11.3	1.3	**-1.29**	**0.15**	**-7.35**	
**24**	**Northern India***	**This study**		**HR L1B-Stacked (This Study)**	**2002 - 2025**	**24**	**1265**	**-27.574**	**3.59**	**-2.176**	**0.28**	**-27.53**	**-39.3%**
		(31)	Global, Ganges River Basin	ITSG-GRACE2016, JPL-M	2003 - 2015	13	1007	**-12.18**	**2.52**	-1.21	0.25	**-15.31**	
		(34)	Ganges River Basin	JPL-M, CSR-M	2002 - 2016	15	**1106**	**-15.81**	**1.61**	-1.43		**-18.09**	
		(29)	Northern India	JPL-M	2002 - 2016	15	1228	-23.30	1.90	**-1.90**	**0.15**	**-24.00**	
		(29)	Eastern India	JPL-M	2002 - 2016	15	664	-19.2	1.1	**-2.89**	**0.17**	**-36.58**	
**25**	**Chotts Basin NWSAS**	**This study**		**HR L1B-Stacked (This Study)**	**2002 - 2025**	**24**	**310**	**-4.48**	**0.89**	**-1.45**	**0.29**	**-4.50**	**-78.2%**
		(35)	NWSAS	JPL-SH, CSR-SH, GFZ-SH, JPL-M, CSR-M	2002 - 2019	18	1000	-3.7	0.12	**-0.37**		**-1.15**	
		(36)	NWSAS	JPL-M, CSR-M	2002 - 2020	18	**1042**	**-3.33**		-0.32	0.02	**-0.99**	

		(37)	NWSAS	JPL-M, CSR-M	2003 - 2016	14	1190	**-3.06**		**-0.26**		**-0.80**	
**26**	**Morocco**	**This study**		**HR L1B-Stacked (This Study)**	**2002 - 2025**	**24**	**136**	**-1.22**	**0.57**	**-0.89**	**0.42**	**-1.21**	**-68.5%**
		(38)	Oum Er-Rbia, Tensift basins	JPL-M, CSR-M, GSFC-M	2002-2022	21	64	**-0.18**	**0.00**	-0.28	0.11	**-0.38**	
**27**	**Saudi Arabia**	**This study**		**HR L1B-Stacked (This Study)**	**2002 - 2025**	**24**	**781**	**-12.72**	**1.65**	**-1.63**	**0.21**	**-12.73**	**-28.8%**
		(39)	Saq Aquifer	JPL-M, CSR-M, JPL-SH, CSR-SH	2002 - 2016	15	**374**	**-2.85**	**0.07**	-1.16	0.02	**-9.06**	
		(29)		JPL-M	2002 - 2016	15	842	-10.5	1.5	**-1.25**	**0.18**	**-9.74**	
**28**	**Fertile Crescent**	**This study**		**HR L1B-Stacked (This Study)**	**2002 - 2025**	**24**	**533**	**-8.58**	**1.45**	**-1.61**	**0.27**	**-8.58**	**-64.0%**
		(40)	Iraq	JPL-M, CSR-M, GSFC-M	2002 - 2020	19				-0.58	0.07	**-3.09**	
**29**	**Western Iran**	**This study**		**HR L1B-Stacked (This Study)**	**2002 - 2025**	**24**	**198**	**-2.92**	**0.78**	**-1.48**	**0.4**	**-2.93**	
**30**	**Southern Iran**	**This study**		**HR L1B-Stacked (This Study)**	**2002 - 2025**	**24**	**360**	**-6.77**	**1.18**	**-1.88**	**0.33**	**-6.77**	**-57.6%**

		(41)	Iran	ITSG-Grace2018	2003 - 2019	17	1700	-12	2	**-0.71**	**0.12**	**-2.54**	
		(31)	Global, Southern Iran	ITSG-GRACE2016, JPL-M	2003 - 2015	13	65	**-0.58**	**0.20**	-0.89	0.3	**-3.20**	
**31**	**NE Iran**	**This study**		**HR L1B-Stacked (This Study)**	**2002 - 2025**	**24**	**334**	**-5.34**	**1.19**	**-1.6**	**0.35**	**-5.34**	**-49.8%**
		(41)	Central Desert, Iran	ITSG-Grace2018	2003 - 2019	17	224	-1.8	0.26	**-0.80**	**0.12**	**-2.68**	
**32**	**SW U.S.**	**This study**		**HR L1B-Stacked (This Study)**	**2002 - 2025**	**24**	471	**-6.75**	**1.29**	**-1.43**	**0.27**	**-6.74**	**-16.8%**
		(42)	U.S., Central Valley (Sacramento + Tulare)	JPL-M, CSR-M	2002 - 2017	15	**155**	-2.00	0.46	-1.29	0.29	**-6.08**	
		(31)	Global, Sacramento Basin	ITSG-GRACE2016, JPL-M	2003 - 2015	13	71	**-0.77**	**0.30**	-1.09	0.42	**-5.13**	
		(29)		JPL-M	2002 - 2016	15	178	-4.2	0.4	**-2.36**	**0.22**	**-11.11**	
**33**	**Southern High Plains**	**This study**		**HR L1B-Stacked (This Study)**	**2002 - 2025**	**24**	**347**	**-5.92**	**1.31**	**-1.71**	**0.38**	**-5.93**	**-23.7%**
		(42)	U.S., High Plains Aquifer	JPL-M, CSR-M	2002 - 2017	15	**203**	-2.62	0.07	-1.29	0.03	**-4.48**	

		(31)	Global, Brazos River Basin		2003 - 2015	13	119	**-1.57**	**0.35**	-1.32	0.29	**-4.58**	
		(29)		JPL-M	2002 - 2016	15	1105	-12.2	3.6	**-1.10**	**0.33**	**-3.83**	
**34**	**Chihuahua Mexico**	**This study**		**HR L1B-Stacked (This Study)**	**2002 - 2025**	**24**	**347**	**-3.79**	**1.13**	**-1.09**	**0.33**	**-3.78**	
**35**	**Central Mexico**	**This study**		**HR L1B-Stacked (This Study)**	**2002 - 2025**	**24**	**161**	**-1.82**	**0.64**	**-1.13**	**0.4**	**-1.82**	**-80.5%**
		(31)			2003 - 2015	13	134	**-0.29**	**0.31**	-0.22	0.23	**-0.35**	
**36**	**Brazilian Highlands**	**This study**		**HR L1B-Stacked (This Study)**	**2002 - 2025**	**24**	**1512**	**-24.3**	**2.48**	**-1.61**	**0.16**	**-24.34**	**-34.4%**
		(31)	Global, Sao Francisco Basin		2003 - 2015	13	637	**-8.85**	**1.78**	-1.39	0.28	**-21.02**	
		(43)	Urucuia Aquifer System	JPL-SH, CSR-SH, GFZ-SH	2003 - 2014	12	**125**	-0.81		-0.65	0.26	**-9.83**	
		(44)	Sao Francisco Basin		2002 - 2017	16	637	**-7.20**	**1.08**	-1.13	0.17	**-17.09**	
				JPL-M	2002 - 2016	15	1132	-16.7	2.9	**-1.48**	**0.26**	**-22.31**	

**37**	**Libya's GMMR**	**This study**		**HR L1B-Stacked (This Study)**	**2002 - 2025**	**24**	**99**	**-0.87**	**0.49**	**-0.88**	**0.49**	**-0.87**	**-73.9%**
		(36)	Murzuk-Djado basin	JPL-M, CSR-M, GSFC-M	2002-2020	28	**489**	**-1.13**		-0.23	0.01	**-0.23**	

Dataset S1 (separate file). TWS trends (2002-2025) at GSFC mascon locations derived from Level-1B regression. Columns provided are as follows:

- latc = Mascon latitude center (degrees), range = -90:90
- lonc = Mascon longitude center (degrees), range = 0.5:359.5
- latw = Mascon latitude width (degrees)
- lonw = Mascon longitude width (degrees)
- areaarc = Mascon area (square arc-degree)
- areaskm = Mascon area (square kilometers)
- loc = Mascon location flag (GIS=1, AIS=3, ice shelf=4, GOA=5, Land=80, Ocean=90)
- regid = Region in this study (NA indicates no assigned region)
- trend = Terrestrial water storage trend (cm water equivalent per year)

SI References

1. H. Dobslaw, *et al.*, A new high-resolution model of non-tidal atmosphere and ocean mass variability for de-aliasing of satellite gravity observations: AOD1B RL06. *Geophys. J. Int.* **211**, 263–269 (2017).

2. Y. Sun, R. Riva, P. Ditmar, Optimizing estimates of annual variations and trends in geocenter motion and J2 from a combination of GRACE data and geophysical models. *J. Geophys. Res. Solid Earth* **121**, 8352–8370 (2016).

3. B. D. Loomis, K. E. Rachlin, D. N. Wiese, F. W. Landerer, S. B. Luthcke, Replacing GRACE/GRACE-FO With Satellite Laser Ranging: Impacts on Antarctic Ice Sheet Mass Change. *Geophys. Res. Lett.* **47**, e2019GL085488 (2020).

4. W. Richard Peltier, D. F. Argus, R. Drummond, Comment on "An Assessment of the ICE-6G_C (VM5a) Glacial Isostatic Adjustment Model" by Purcell et al. *J. Geophys. Res. Solid Earth* **123**, 2019–2028 (2018).

5. S.-C. Han, R. Riva, J. Sauber, E. Okal, Source parameter inversion for recent great earthquakes from a decade-long observation of global gravity fields. *J. Geophys. Res. Solid Earth* **118**, 1240–1267 (2013).

6. B. D. Loomis, D. Felikson, T. J. Sabaka, B. Medley, High-Spatial-Resolution Mass Rates From GRACE and GRACE-FO: Global and Ice Sheet Analyses. *J. Geophys. Res. Solid Earth* **126**, e2021JB023024 (2021).

7. A. N. Tikhonov, Solution of incorrectly formulated problems and the regularization method. *Sov. Math Dokl* **4**, 1035–1038 (1963).

8. B. D. Loomis, *et al.*, Water Storage Trends in High Mountain Asia. *Front. Earth Sci.* **7**, 235 (2019).

9. B. D. Loomis, S. B. Luthcke, T. J. Sabaka, Regularization and error characterization of GRACE mascons. *J. Geod.* **93**, 1381–1398 (2019).

10. D. K. Hall, B. D. Loomis, N. E. DiGirolamo, B. A. Forman, Snowfall Replenishes Groundwater Loss in the Great Basin of the Western United States, but Cannot Compensate for Increasing Aridification. *Geophys. Res. Lett.* **51**, e2023GL107913 (2024).

11. M. C. Peel, B. L. Finlayson, T. A. McMahon, Updated world map of the Köppen-Geiger climate classification. *Hydrol. Earth Syst. Sci.* **11**, 1633–1644 (2007).

12. J. W. Lichstein, T. R. Simons, S. A. Shriner, K. E. Franzreb, Spatial Autocorrelation and Autoregressive Models in Ecology. *Ecol. Monogr.* **72**, 445–463 (2002).

13. J. Durbin, Estimation of Parameters in Time-Series Regression Models. *J. R. Stat. Soc. Ser. B Methodol.* **22**, 139–153 (1960).

14. M. Lou, H. Zhang, X. Lei, C. Li, H. Zang, Spatial Autoregressive Models for Stand Top and Stand Mean Height Relationship in Mixed Quercus mongolica Broadleaved Natural Stands of Northeast China. *Forests* **7**, 43 (2016).

15. M. Koley, A. K. Bera, To use, or not to use the spatial Durbin model? – that is the question. *Spat. Econ. Anal.* **19**, 30–56 (2024).

16. J. J. Moré, The Levenberg-Marquardt algorithm: Implementation and theory in *Numerical Analysis*, G. A. Watson, Ed. (Springer, 1978), pp. 105–116.

17. C. G. Thompson, R. S. Kim, A. M. Aloe, B. J. Becker, Extracting the Variance Inflation Factor and Other Multicollinearity Diagnostics from Typical Regression Results. *Basic Appl. Soc. Psychol.* **39**, 81–90 (2017).

18. S.-I. Lee, Developing a bivariate spatial association measure: An integration of Pearson's r and Moran's I. *J. Geogr. Syst.* **3**, 369–385 (2001).

19. P. A. P. Moran, The Interpretation of Statistical Maps. *J. R. Stat. Soc. Ser. B Methodol.* **10**, 243–251 (1948).

20. K. Winkler, R. Fuchs, M. Rounsevell, M. Herold, Global land use changes are four times greater than previously estimated. *Nat. Commun.* **12**, 1–10 (2021).

21. B. Lehner, *et al.*, Global Reservoir and Dam Database, Version 1 (GRanDv1): Reservoirs, Revision 01. *NASA Socioecon. Data Appl. Cent. SEDAC* (2011). https://doi.org/https://doi.org/10.7927/H4HH6H08.

22. S. Siebert, V. Henrich, K. Frenken, J. Burke, Global Map of Irrigation Areas version 5. *Rheinische Friedrich-Wilhelms-Univ. Bonn Ger. Food Agric. Organ. Unite Nations Rome Italy* (2013). Available at: https://www.fao.org/aquastat/en/geospatial-information/global-maps-irrigated-areas/latest-version/ [Accessed 22 April 2022].

23. J. M. Salmon, M. A. Friedl, S. Frolking, D. Wisser, E. M. Douglas, Global rain-fed, irrigated, and paddy croplands: A new high resolution map derived from remote sensing, crop inventories and climate data. *Int. J. Appl. Earth Obs. Geoinformation* **38**, 321–334 (2015).

24. H. Hersbach, *et al.*, ERA5 monthly averaged data on single levels from 1940 to present. *Copernic. Clim. Change Serv. C3S Clim. Data Store CDS* (2023). https://doi.org/10.24381/cds.f17050d7.

25. R. Hugonnet, *et al.*, Accelerated global glacier mass loss in the early twenty-first century. *Nat. 2021* **592**, 726–731 (2021).

26. D. N. Wiese, D.-N. Yuan, C. Boening, F. W. Landerer, M. M. Watkins, JPL GRACE mascon ocean, ice, and hydrology equivalent water height release 06 coastal resolution improvement (CRI) filtered version 1.0 Ver. 1.0. *PO.DAAC* (2018). https://doi.org/http://dx.doi.org/10.5067/TEMSC-3MJC6.

27. H. Save, S. Bettadpur, B. D. Tapley, High-resolution CSR GRACE RL05 mascons. *J. Geophys. Res. Solid Earth* **121**, 7547–7569 (2016).

28. A. Kvas, *et al.*, GOCO06s- A satellite-only global gravity field model. *Earth Syst. Sci. Data* **13**, 99–118 (2021).

29. M. Rodell, *et al.*, Emerging trends in global freshwater availability. *Nature* **557**, 651–659 (2018).

30. Abhishek, T. Kinouchi, R. Abolafia-Rosenzweig, M. Ito, Water Budget Closure in the Upper Chao Phraya River Basin, Thailand Using Multisource Data. *Remote Sens.* **14**, 173 (2022).

31. B. D. Vishwakarma, P. Bates, N. Sneeuw, R. M. Westaway, J. L. Bamber, Re-assessing global water storage trends from GRACE time series. *Environ. Res. Lett.* **16**, 034005 (2021).

32. J. Liu, *et al.*, Impacts of water resources management on land water storage in the North China Plain: Insights from multi-mission earth observations. *J. Hydrol.* **603**, 126933 (2021).

33. W. Qu, P. Zhang, P. Chen, J. Li, Y. Gao, Spatiotemporal Variations and Sustainability Characteristics of Groundwater Storage in North China from 2002 to 2022 Revealed by GRACE/GRACE Follow-On and Multiple Hydrologic Data. *Remote Sens.* **16**, 1176 (2024).

34. S. Dangar, V. Mishra, Natural and anthropogenic drivers of the lost groundwater from the Ganga River basin. *Environ. Res. Lett.* **16**, 114009 (2021).

35. M. Ahmed, Sustainable management scenarios for northern Africa's fossil aquifer systems. *J. Hydrol.* **589**, 125196 (2020).

36. B. R. Scanlon, *et al.*, Linkages between GRACE water storage, hydrologic extremes, and climate teleconnections in major African aquifers. *Environ. Res. Lett.* **17**, 014046 (2022).

37. F. Frappart, Groundwater Storage Changes in the Major North African Transboundary Aquifer Systems during the GRACE Era (2003–2016). *Water 2020 Vol 12 Page 2669* **12**, 2669 (2020).

38. H. Ouatiki, A. Boudhar, M. Leblanc, Y. Fakir, A. Chehbouni, When climate variability partly compensates for groundwater depletion: An analysis of the GRACE signal in Morocco. *J. Hydrol. Reg. Stud.* **42**, 101177 (2022).

39. A. Mohamed, K. Abdelrahman, A. Abdelrady, Application of Time-Variable Gravity to Groundwater Storage Fluctuations in Saudi Arabia. *Front. Earth Sci.* **10** (2022).

40. A. Othman, A. Abdelrady, A. Mohamed, Monitoring Mass Variations in Iraq Using Time-Variable Gravity Data. *Remote Sens.* **14**, 3346 (2022).

41. P. Saemian, M. J. Tourian, A. AghaKouchak, K. Madani, N. Sneeuw, How much water did Iran lose over the last two decades? *J. Hydrol. Reg. Stud.* **41**, 101095 (2022).

42. B. R. Scanlon, *et al.*, Effects of climate and irrigation on GRACE-based estimates of water storage changes in major US aquifers. *Environ. Res. Lett.* **16**, 094009 (2021).

43. R. D. Gonçalves, R. Stollberg, H. Weiss, H. K. Chang, Using GRACE to quantify the depletion of terrestrial water storage in Northeastern Brazil: The Urucuia Aquifer System. *Sci. Total Environ.* **705**, 135845 (2020).

44. C. E. Ndehedehe, V. G. Ferreira, Assessing land water storage dynamics over South America. *J. Hydrol.* **580**, 124339 (2020).
